# Shaping the Organ: A Biologist Guide to Quantitative Models of Plant Morphogenesis

**DOI:** 10.3389/fpls.2021.746183

**Published:** 2021-10-05

**Authors:** Marco Marconi, Krzysztof Wabnik

**Affiliations:** Centro de Biotecnología y Genómica de Plantas, Universidad Politécnica de Madrid (UPM) - Instituto Nacional de Investigación y Tecnología Agraria y Alimentaria (INIA), Pozuelo de Alarcón (Madrid), Spain

**Keywords:** plant morphogenesis, computational modeling, plant development, L-systems, finite element method, position based dynamics, cell complex

## Abstract

Organ morphogenesis is the process of shape acquisition initiated with a small reservoir of undifferentiated cells. In plants, morphogenesis is a complex endeavor that comprises a large number of interacting elements, including mechanical stimuli, biochemical signaling, and genetic prerequisites. Because of the large body of data being produced by modern laboratories, solving this complexity requires the application of computational techniques and analyses. In the last two decades, computational models combined with wet-lab experiments have advanced our understanding of plant organ morphogenesis. Here, we provide a comprehensive review of the most important achievements in the field of computational plant morphodynamics. We present a brief history from the earliest attempts to describe plant forms using algorithmic pattern generation to the evolution of quantitative cell-based models fueled by increasing computational power. We then provide an overview of the most common types of “digital plant” paradigms, and demonstrate how models benefit from diverse techniques used to describe cell growth mechanics. Finally, we highlight the development of computational frameworks designed to resolve organ shape complexity through integration of mechanical, biochemical, and genetic cues into a quantitative standardized and user-friendly environment.

## Introduction

Plant development has inspired the interest of scientific minds since antiquity. The first attempt to formulate plant growth into a mathematically coherent framework was given by Thompson (1917) in his landmark book *On Growth and* Form. Idea of Thompson (1917) was that morphogenesis could be summarized as a series of coherent geometrical transformations leading to the spacious diversity of biological forms. The concept of morphogenesis is therefore quite general; we usually define morphogenesis as a recipe to build an organism with elements such as individual cells, genes products and biochemical signals. While many cells proliferate to recreate the organism’s adult shape some of them may differentiate into specialized tissues. Understanding the rules behind this decision-making process is at the core of organ patterning. To grasp the principles of morphogenesis, one has to consider different scales of organization such as growth mechanics, biochemical reactions, and genetic blueprint. Given the sheer number of elements involved in a morphogenetic pattern formation (i.e., the plant embryo may contain tenths of cells), it is impractical to analyze it without the support from computational tools.

In biology, computer models of patterning made their appearance in the 1960s with the widespread use of computer algorithms. Most of these models were based on static templates and did not include cell growth dynamics (Turing, 1952). Remarkably, Ulam (1962) showed that incredibly complicated forms and structures could be generated using cellular automata. Over the years, many researchers have recognized the importance of positional information in morphogenesis, as growth and cell division affect chemical gradients by diluting and degrading biological molecules (Wolpert, 1969). One of the earliest computational models of expanding plant tissue was proposed by Korn (1969). This model simulates the expansion of the alga *Coleochaete scutata* by applying a series of rules for cell growth and division over a hexagonal lattice (Korn, 1969). A similar work on two-dimensional growth of cell populations was previously introduced by Eden (1961). Pattern generation was a popular topic in computer science in that period and researchers were eager to find applications in biology and other fields. Cohen (1967) published a computer program capable of producing branching patterns reminiscent of leaf vascularization. These initial attempts to represent descriptive growth were not formalized into a comprehensive computational framework until the introduction of L-systems by Lindenmayer (1968). L-systems quickly gained popularity in the 1970s and until today are one of the favored methodologies for modeling plant architectures. Similar approaches to L-systems were developed for models of tree-like branching formation (Honda, 1971, 1983). Simultaneously, early applications of continuous mechanics methods [i.e., finite element method (FEM)] were applied to green algae development (Niklas, 1977). These were followed by the implementation of anisotropic deformation and stress–strain relation in models of plant growth (Silk and Erickson, 1979; Hejnowicz and Romberger, 1984; Peters and Tomos, 1996). Furthermore, the relentless increase of computational power of modern machines allowed the definition of increasingly complex structures, such as tissues and entire organs (Glazier and Graner, 1993).

These initial successes in modeling plant development motivated the incorporation of biomechanics properties to further increase the realism of plant form generation. Lockhart (1965) proposed a model to predict cell wall growth rate from internal turgor pressure by formulating a set of rules regulating the behavior of an idealized cell wall, which will later become the *de facto* standard for mechanical modeling of plant cell elongation. However, these formulas could not represent important mechanical aspects exhibited by living cells such as water transpiration, wall stress relaxation, pressure relaxation, and elastic deformations (Geitmann and Ortega, 2009), which were addressed by follow-up studies: Cosgrove (1986) extended Lockhart’s paradigm (which only applied to single cells in isolation) to multicellular organization; Silk and Wagner (1980) proposed a non-compartmented continuum model; Ortega (1985) augmented Lockhart’s equation with an elastic component, while Veytsman and Cosgrove (1998) rederived the formula in terms of thermodynamics of polymer networks. These studies greatly improved Lockhart’s initial idea and transformed it into a more flexible framework suited for complex environments.

In the last decade, we have witnessed an exponential growth of theoretical and experimental studies incorporating either biochemical (Leyser, 2018) or biomechanical (Kierzkowski and Routier-Kierzkowska, 2019) aspects of plant organ growth. The continuous feedback between these two signals can potentially give rise to outcomes hardly predictable through simple human intuition, and thus safeguarding the necessity for even more advanced computational modeling techniques. To guarantee reproducibility and standardization, several pre-packaged and ready-to-use modeling frameworks have been developed (Prusinkiewicz et al., 2000; Pradal et al., 2008; Merks et al., 2011) which provide an interactive environment for biologists lacking programming knowledge.

In this review, we evaluate common biological issues and bottlenecks in modeling plant organ forms as well as their implications with respect to model realism. Finally, we present some of the most popular modeling frameworks that have been developed in the attempt to solve these issues.

## Grasping on the Complexity of Plant Organ Structure

In general, computational models are designed to serve two main purposes: describing a natural phenomenon of interest to gain insight into the mechanics of the process or to make predictive statements about an idealized hypothesis to forecast the result of *in vivo* experiments. There are several recurring questions in plant growth and morphogenesis that have been under the scrutiny of researchers for a long time and that could be now addressed with quantitative computer models.

**Figure 1:**
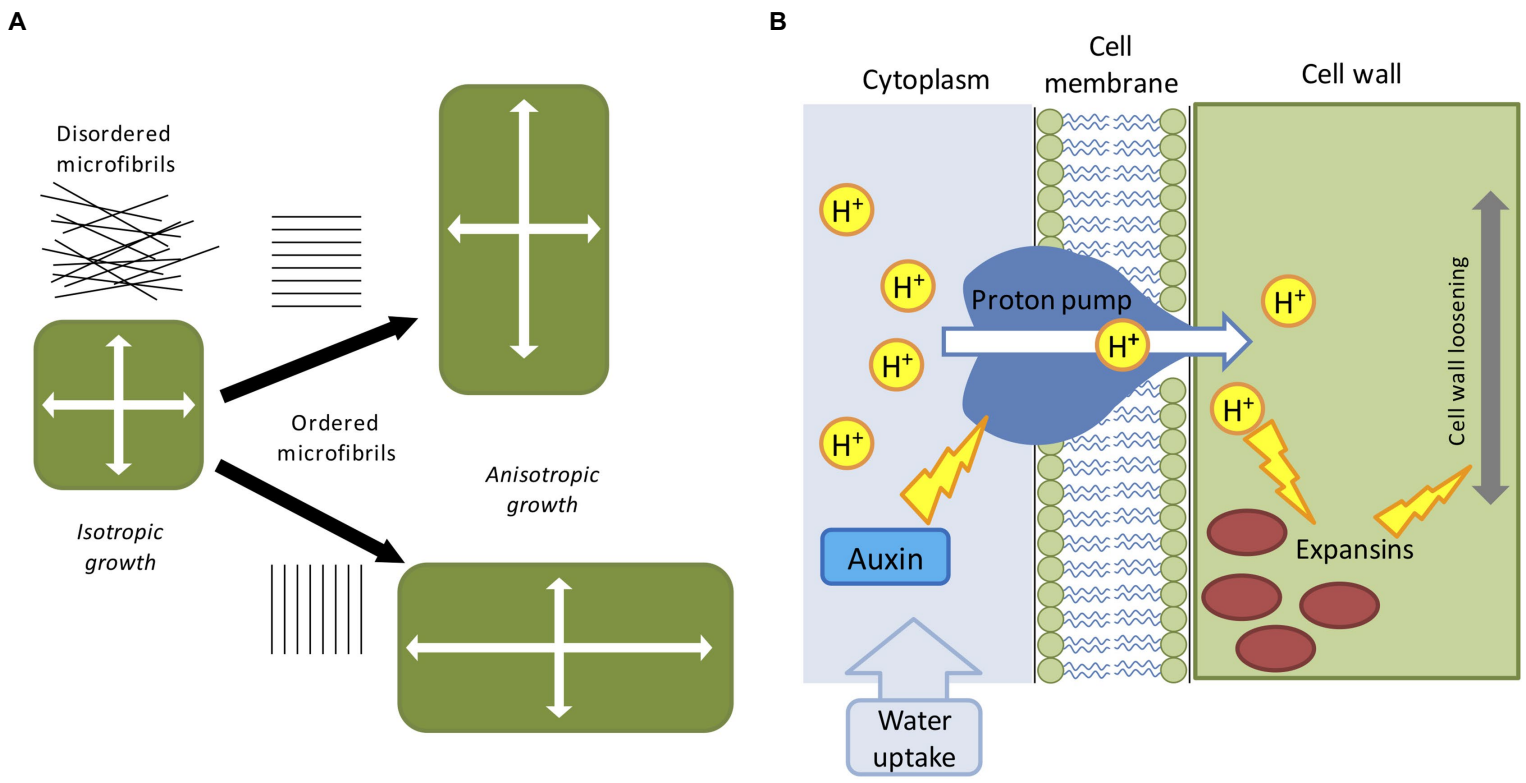
Mechanical and biochemical cues in computer models of plant development. **(A)** The direction of maximal expansion in plant cells depends on the orientation of cellulose microfibrils. Disordered microfibrils cause the isotropic all-around growth. A globally ordered structure of cellular microfibrils determines the anisotropic elongation, such that the growth direction is biased. **(B)** The acid-growth hypothesis; auxin promotes the proton pump activity which then acidifies the cell wall through the extrusion of H^+^ into the apoplast. Acidification promotes expansins and other cell wall-related enzymes, leading to the relaxation of the cell wall material, and successive cell growth.

Plants possess apical/basal polarity axes (Jürgens, 2001). Single cells can expand either isotropically or anisotropically (Figure 1A), and cell polarity contributes to the collective choice of single cells which eventually determine the future shape of the plant (Wabnik et al., 2013). Some plant organs such as the root, root hairs, fruit, stems, and pollen tubes display a clearly anisotropic shape (Baskin, 2005). Growth anisotropy correlates with the direction of cellulose microfibrils deposited during cell wall synthesis (Li et al., 2014), but the exact mechanisms of how anisotropic growth is achieved are largely unknown. Cellulose fibers are very stiff and can be commonly found disordered or oriented along a preferred cell axis, as a result the cell wall remains stiff along a specific direction (Cosgrove, 2005). Microfibrils are often parallel to cortical microtubule orientation and it is thought that cellulose microfibril synthesis is guided by cortical microtubule tracks (Paradez et al., 2006). It has been shown that cortical microtubules align with maximal tensile stress in plant tissues (Hamant et al., 2019), indicating the existence of a feedback loop which reinforces the cell anisotropy against principal stress directions (Figure 1A). Surprisingly, it has been suggested that even isotropic growth could generate anisotropic patterns as a result of differential growth rates between adjacent tissues (Kennaway et al., 2011; Marconi et al., 2021). Other studies have also hypothesized the existence of morphogen-driven growth polarity fields (Mansfield et al., 2018).

The factors partially involved in anisotropic growth are multiple and are not limited to cytoskeleton configuration. Cell elongation is mainly driven by two mechanisms: cell wall deformation by internal turgor pressure and cell wall growth mediated by enzymatic reactions [involving expansins, xyloglucan endotransglucosylase/hydrolase (XTH), and pectin-modifying enzymes (PMEs)] and favored by hormones accumulation (like auxin, gibberellins and abscisic acid; Cosgrove, 2016; Smithers et al., 2019). The latter is commonly known as the acid growth hypothesis (Figure 1B; Rayle and Cleland, 1992). Turgor pressure is created by the continuous uptake of water from the external environment, which in turn exerts physical stresses on the cell wall (Cosgrove, 1993). The cell wall is incredibly rigid, capable of sustaining extremely high internal pressure (up to three atmospheres), and it is generally regarded as a viscoelastic material (Geitmann and Ortega, 2009). For the sake of simplicity, turgor pressure can be considered constant during cell growth (Schopfer, 2006), and below a certain level of deformation the cell behaves as an elastic material and slowly return to its initial state (Proseus et al., 1999). In case of irreversible deformation, the cell would acquire a new shape assisted by the enzymatic effect which replenishes the wall with new structural materials (Dumais et al., 2006). Moreover, plant cells are bound together by the cell wall, meaning that, contrary to animal cells, they cannot slide along each other or migrate to other regions. A consequence of this feature is that plant tissues are very rigid and single cell movements are transmitted in cascade to neighboring cells creating a tissue-spanning mechanical stress (Hejnowicz et al., 2000). As mentioned earlier, the first attempt to derive a set of mathematical equations able to describe mechano-hydraulic cell growth was proposed by Lockhart (1965), which stated that cell expansion rate is a function of cell volume, cell wall extensibility, turgor pressure, and turgor yield threshold. This equation represented the first attempt to couple water uptake and cell wall mechanics, where irreversible cell expansion is driven by the action of internal turgor pressure (Lockhart, 1965). However, the role of water fluxes is neglected in the majority of plant models, by simply assuming that the turgor pressure is a constant driving force. This unrealistic assumption is acceptable for single cell experiments but presents some practical problems in a multicellular context: neighboring cells grow at different rates (Hong et al., 2018) and water flow is affected by the relative geometry of the interconnected cells (Dinneny, 2020) as water availability is not uniform along the tissue (Robbins and Dinneny, 2018). Moreover, growth rate and pressure level are not always correlated even in single cells (Rojas and Huang, 2018). To reconcile Lockhart’s equation with these issues and generalize it to a multicellular environment, a recent study proposed a mechanohydraulic model (Long et al., 2020), where cell growth and turgor pressure can autonomously emerge from the interaction of tissue mechanics and tissue hydraulics. Using atomic force microscopy the authors showed significant variability in turgor pressure between cells (Long et al., 2020). Smaller cells resulting from cell division presented higher internal turgor press, while larger cells had lower pressure. In a situation where cell division does not occur, cells growth rate would tend to homogenize with turgor pressure decreasing as cell size increase (Long et al., 2020). Overall, despite its shortcomings Lockhart’s equation is still today the base model for many applications in cell physiology.

**Figure 2:**
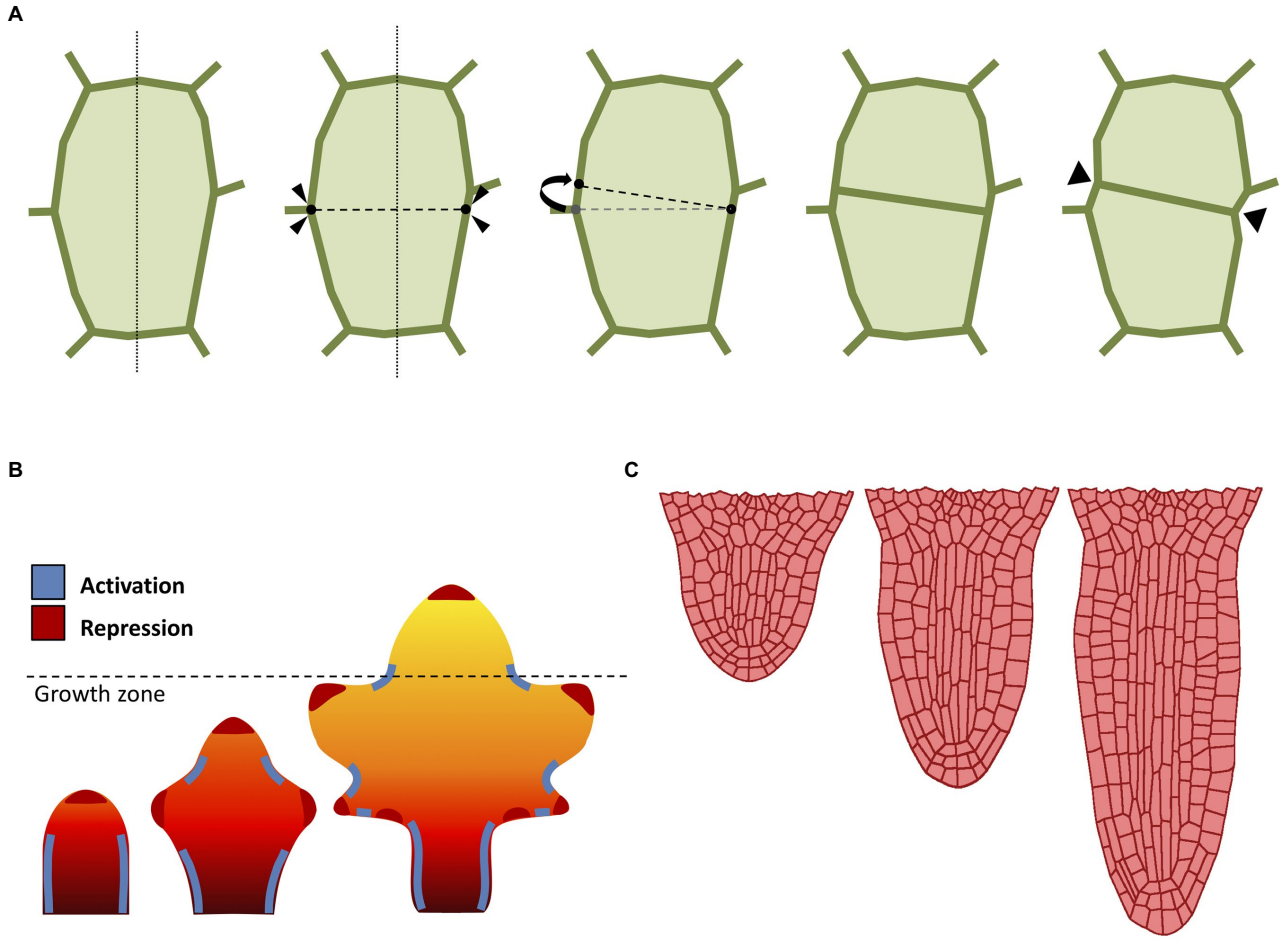
Computational approaches to cell division and growth. **(A)** A popular implementation of cell division rule. The cell wall is represented as a polygon of vertices connected by edges. According to the shortest-distance rule (Errera, 1888), the cell is divided by identifying the shortest division plane cutting through the cell. To avoid conflict with existing vertices, the two ends of the division plane can be spread apart by a minimal threshold distance. Cell division splits the cell into two daughter cells yielding two new connected vertices in the cell wall. Often, the cell wall is “pinched” along the newly created vertices (Smith et al., 2006b). **(B)** Computational model of leaf shape development (adapted from Runions and Tsiantis, 2017). Mature leaf shape is achieved through the interaction between three components: a proximal-distal hypothetical morphogen and two master regulators. The basipetal red-orange-yellow gradient region defines the action of the growth morphogen (red: higher growth, yellow: lower growth), where the dashed line indicates the border between the actively growing region and the differentiation zone. The marginal patterning of the leaf blade is the result of the combined action of a local growth activator (red) and a growth suppressor (blue). **(C)** Computational model of radicle emergence (adapted from Marconi et al., 2021). This simulation reproduces the embryonic emergence of the root meristem of *Arabidopsis*. Organ growth follows the combined action of cell elongation, cell division, and tissue mechanics. Note that the organ maintains a distinct anisotropic form through the self-organization of cortical microtubules despite each single cell being expanded by uniform turgor pressure (see Marconi et al., 2021 for details).

A major technical issue when dealing with growing organ is how to approach cell division (Figure 2A). When considering the organ as a continuous form without single-cell identities (i.e., FEM methods), cell division is typically omitted and organ growth is usually obtained through iterations of tissue remeshing and growth cycles. The problem of cell division rules had sparked interest since the nineteenth century, when Herrera proposed the so-called shortest wall solution (Errera, 1888). In 2D models, cells are commonly represented as polygons, so Herrera’s rule can be implemented by simply using as division plane the shortest possible edge that divides this polygon in half (Figure 2A). The evidence seems to suggest that cell division does not occur at random (Sahlin and Jönsson, 2010), and that each cell locally controls its own division rules (Besson and Dumais, 2011). Several studies have also confirmed the existence of strong genetic control over cell division geometry (Dong et al., 2009). In plants, there is a strong correlation between the division plane and microtubule orientation (de Keijzer et al., 2014). Therefore, it has been proposed that cell divides orthogonal to the principal direction of growth where the maximal tensile stress occurs (Louveaux et al., 2016).

The combined action of cell growth and division gives rise to different cellular patterns and eventually determines the global organ shape. Even the simplest multicellular organisms may exhibit complex cellular patterning (Dupuy et al., 2010). Plants produce diverse geometric shapes, such as flowers, leaves, and networks of roots. A prominent example of patterning in plants is phyllotaxis; the process in which leaves (but also flowers and petals) are arranged around the growing vegetative stems. The beautiful geometry of phyllotaxis has attracted the attention of botanists and mathematicians since ancient times (Adler et al., 1997), and the process is known to be driven by the interaction between the phytohormone auxin and tissue growth to optimize light capture (Strauss et al., 2020). Phyllotaxis has been the subject of many attempts to formalize its mechanism through computational modeling techniques (Mitchison, 1977; Jönsson et al., 2006; Smith et al., 2006a; Zhang et al., 2021).

Morphogenesis in plants is not limited to global multicellular interplay, as whole organs are able to direct growth to form shapes designed to accomplish a specific function or to better adapt to the environment. Leaves in particular are known to exist in a plethora of shapes and dimensions, despite their almost indistinguishable primordia (Vlad et al., 2014; Kierzkowski et al., 2019). Leaves emerge from the shoot apical meristem as a result of the phyllotactic process described above, and their patterning is regulated by the phytohormone auxin (Bayer et al., 2009), which promotes growth and differentiation through the formation of maxima along the leaf margins (Figure 2B). Nutrients are carried along the leaf surface throughout the venation system, and the chaotic distribution of this network of veins markedly contrasts with the pristine symmetrical beauty of other processes like phyllotaxis. Nonetheless, researchers managed to devise models of venation, where the apparent complexity of these patterns is simply the result of a self-organizing process of continuous leaf surface “colonization” driven by the tandem action of auxin and cellular growth (Runions et al., 2005). Several other computational models have been developed to understand the diversification of leaf geometry (Lindenmayer, 1977; Rolland-Lagan et al., 2005; Kierzkowski et al., 2019).

Plants also possess radially symmetric organs such as the primary root (Figure 2C). This rod-shaped organ has the main purpose of penetrating through soil in search of nutrients, as well as providing anchoring and stability to the plant (Petricka et al., 2012). The typical *Arabidopsis* root is an anisotropic structure made of radially distributed cells, with different tissues longitudinally oriented and clearly distinguishable under the microscope (Dolan et al., 1993). The root meristem in particular displays a surprisingly conserved cellular organization, with each cell type occupying a defined position and playing a determined role during cell proliferation (Van Norman, 2016). Root growth is regulated by auxin which flows through the inner tissues toward the tip, where it accumulates creating a maximum, and it is later refluxed back through the outer tissues (Kepinski and Leyser, 2005). The mechanisms behind root growth have been studied and tested *in-silico* using several computational models (Grieneisen et al., 2007; Fozard et al., 2013; Bassel et al., 2014; Jensen and Fozard, 2015; Marconi et al., 2021).

We have provided examples of complex plant organ shapes found in nature and in the following sections, we will present various modeling methodologies developed to address organ shape complexity. A comprehensive model of morphogenesis requires the definition of a digital structure underlying the biological tissue of interest as well as a set of biologically sound rules for growth and patterning.

## Digital Representation of Plant Tissues

The first problem to consider when modeling morphogenetic processes in plants is the digital representation of the underlying plant organ geometry. Whereas biological tissues are inherently continuous, the internal computer memory only allows discrete elements. The vast majority of models described in the literature typically rely on several “digital” paradigms. However, as memory and computational power are limited, tissue topology representations must balance model performance with model accuracy. The “digital” representations are concerned with a representation of plant tissue topology amenable to user interaction by providing both ease of use (sometimes with a graphical user interface), and flexibility, allowing the user to control complex biological processes such as growth and cell proliferation.

### Lattice-based and Particle-based Representations

**Figure 3:**
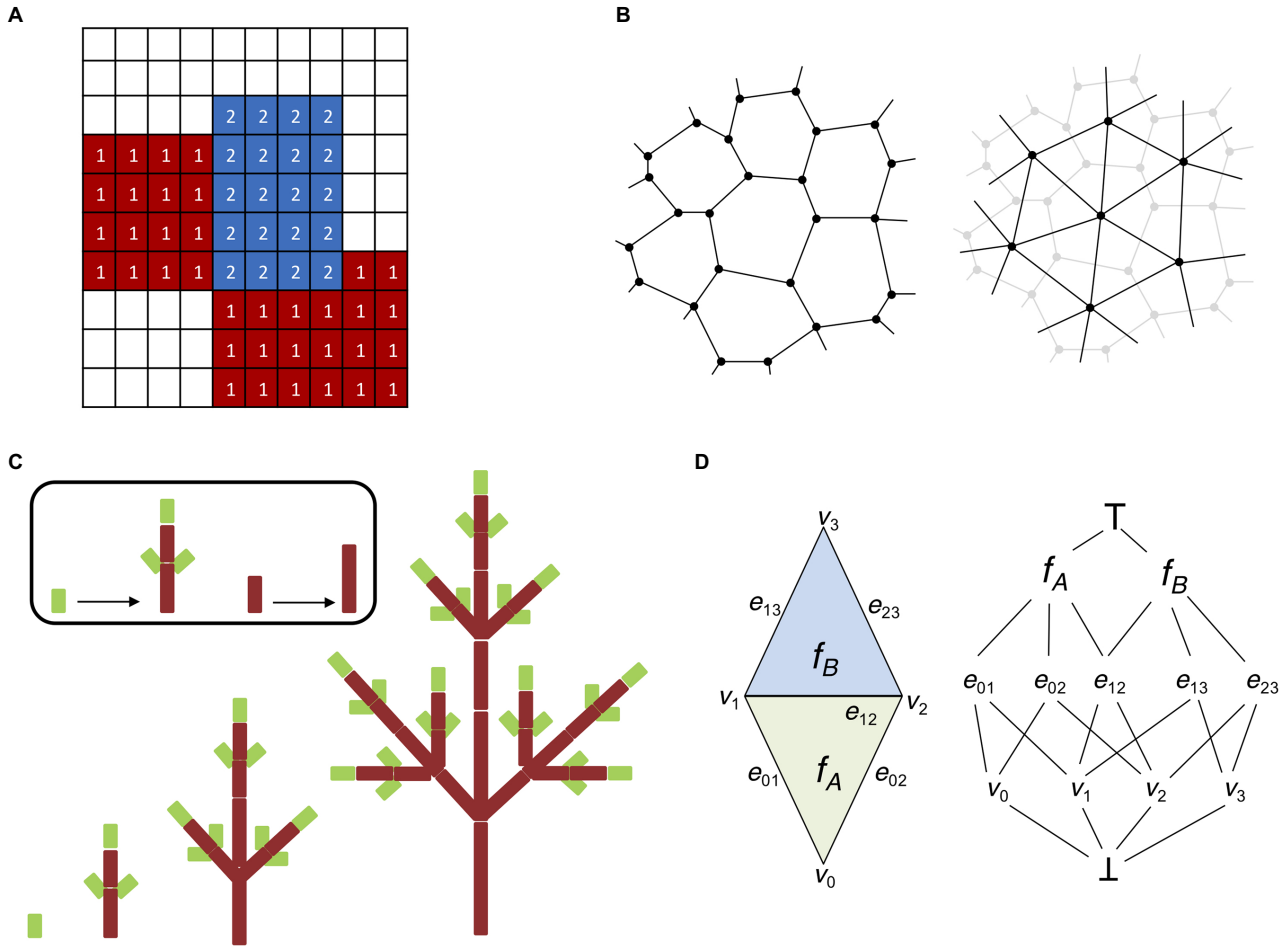
Digital representations of plant tissues. **(A)** Lattice grid representing a basic cellular potts model (CPM). Each element (or pixel) of the grid possesses a specific identity; in this case, we have two cellular types (indicated by 1 and 2) and a medium (no labeling). CPMs are solved by minimizing the Hamiltonian energy of the system, which allows the elements of each cellular type to “group together” and isolate from the surrounding medium. **(B)** Two possible ways to represent multicellular tissues with vertex-based models. Cells can be identified by their cell walls (left). Cell walls are represented as polygons made of vertices connected by edges shared with adjacent cells (in accordance with the biological properties of plant cell walls). Cells can be identified by their centroid (right). Cells are represented by a network of vertices and edges connecting the centroids of adjacent cells. Cell walls and boundaries can be abstracted in several ways, i.e., using Voronoi partitioning (Mosca et al., 2018). **(C)** L-systems modeling (adapted from Prusinkiewicz et al., 2018). An idealized realization of an L-system simulation is shown. The different stages of development of a simple tree branching structure are obtained from simple axioms and recursively applying procedural rules to a small group of elements. **(D)** Schematic representation of the “cell complex.” A simple two-dimensional cell complex made of two connected triangles (left) can be represented as an incident graph (right). Notice how the three cell dimensions (vertices, edges, and faces) occupy three different levels and share boundaries with each other. There are also two pseudocells _┴_ and ^┬^ as the infimum and supremum of the incidence graph (see Prusinkiewicz and Lane, 2013 for details).

One of the main issues faced when embarking on the challenging problem of modeling multi-cellular organisms is the level of cell structure abstraction. Large tissues composed of thousands of cells can be very computationally demanding (Krul et al., 2003), and some form of simplification is often unavoidable. Lattice-based models are a derivation of the notion of cellular automata initially proposed by Ulam (1962), where cell-to-cell interactions are regulated by transition functions dependent on the current state of the cell and its neighbors. In lattice-based models each cell responds independently to external stimuli and follows cell-specific rules (Hwang et al., 2009). A common extension of lattice-based models suitable for modeling tissues is the cellular potts model (CPM; Glazier and Graner, 1993). A CPM is composed of a grid-like lattice where each site is denoted as a “pixel” possessing different identities, such as the cell or the medium (Figure 3A). Cells are allowed to grow, divide, and interact with each other, and the output of a CPM results from the interaction of the collective behavior of individual entities and not from global rules acting on the whole system (Voss-Böhme, 2012).

Cellular potts models use an energy-based approach to simulate growth by minimizing the total energy of the system. A main concern with CPM is their inability to properly reproduce the effect of the plant cell wall (Mele et al., 2015), but a solution to this problem was proposed with the formulation of hybrid mechanical systems (Merks et al., 2011). CPMs have been previously used to model auxin dynamics in the growing root of *Arabidopsis thaliana* (Grieneisen et al., 2007; Laskowski et al., 2008; Mähönen et al., 2014), leaf venation and meristem development (Wabnik et al., 2010; Merks et al., 2011), shoot apical meristem generation and maintenance (Banwarth-Kuhn et al., 2019), vascular tissue formation (Banwarth-Kuhn et al., 2019), and division plane selection in *A. thaliana* (Moukhtar et al., 2019). Also, non-CPM lattices have been employed to model the *Arabidopsis* root (Mironova et al., 2010).

Analogous to lattice-based models are particle-based models (MacAl and North, 2010), where each element (usually cells) is represented as a single particle connected to other particles by permanent bonds. The tissue system is updated by steepest descent minimization which relaxes the forces between the particles. Models of plant tissues implemented with particle systems have been mostly applied to patterning static non-growing tissues (Deinum et al., 2017; Chakrabortty et al., 2018; Mirabet et al., 2018) or agent-based modeling of auxin transport dynamics (Garnett et al., 2010), but they can also be in principle extended to simulate both growth and cell division (Van Opheusden and Molenaar, 2018).

### Vertex-Based Graphs

From the onset of embryonic development up until the final adult stage, plant tissues are subject to the effect of internal and external forces (Ten Hove et al., 2015). Internal forces are the result of cell expansion and proliferation, mostly driven by the action of turgor pressure (Shabala and Lew, 2002). External forces instead result from gravity and the interaction with the environment (Masson et al., 2002). Plant cells are interconnected by a common structure known as the cell wall, which means that unlike animal cells they are not allowed to freely dislodge from their current position and independent mechanical movement is highly constrained (Liepman et al., 2010). A deeper understanding of tissue morphogenesis requires taking into account the laws of mechanics acting on the cell wall. Internal and external forces induce cell wall deformation, which is difficult to model without cellular-level abstraction. Vertex models were proposed to investigate the rules governing cell motility and cell–cell interactions during morphogenesis (Weliky et al., 1991). In a vertex model, cell boundaries are represented by a network of vertices interconnected by edges (Davidson et al., 2010). Therefore, a single cell is rendered as a polygon, where each edge represents the cell wall shared with a neighboring cell or the external space (Figure 3B). The mechanical properties of a vertex model are usually implemented on top of the vertex-edge graph as a simple mass–spring system, where cell growth is driven by updating the resting length of the edge springs (de Boer et al., 1992). Vertex models can also include advanced formulations of the Newtonian laws of motion to create more flexible applications (Fletcher et al., 2014), and they can be easily extended to simulate 3D structures (Alt et al., 2017). Vertex models have been used as the theoretical foundation for the Vertex-Vertex (VV) system (Smith, 2006). The VV system describes a methodology for modeling dynamical surfaces on a discrete 2D manifold topology, which generalizes vertex models to make them practical in a wide variety of situations. The VV system has been released with a ready-to-use implementation in C++, and successfully used in a range of applications, such as phyllotaxis (Smith et al., 2006a; Smith and Bayer, 2009; Hartmann et al., 2019), lateral root response to gravitropism (Waidmann et al., 2019), establishment of apical-basal axis in the plant embryo (Wabnik et al., 2013), leaf shape development (Kierzkowski et al., 2019), leaf venation patterns (Runions et al., 2005), tissue cell polarity establishment (Abley et al., 2013), cells shape lobbiness (Sapala et al., 2018), root growth on nutrient availability (Ötvös et al., 2021), and lateral root priming (Perianez-Rodriguez et al., 2021).

### L-Systems

The need for models capable of describing elaborate morphogenetic processes in flowering plants prompted many researchers to focus on models capable of producing self-generating structures. Lindenmayer (1968) proposed a formal grammar system inspired by cellular automata the L-systems (Prusinkiewicz et al., 2018). L-systems are descriptive models based on natural language processing that simulate plant growth and organ development by assuming an initial set of symbols and production rules that can recursively expand the original set into more and more complex fractal-like structures (Figure 3C). Contrary to cellular automata, which deploy a space-centered Eulerian perspective, L-systems aim at establishing a structure-focused Lagrangian view (Prusinkiewicz and Runions, 2012; Prusinkiewicz et al., 2018). The modular structure of plants therefore represents the perfect application ground for the L-system approach.

L-systems are versatile parametric models that allow the incorporation of molecular-level processes and genetic regulatory networks, and they have been used to recreate the vegetative development of simple multicellular organisms like *Anabaena* (Lindenmayer, 1975) or complex forms such as trees (Allen et al., 2005). This technique has been successfully applied to important research topics in plant development including phyllotactic patterning (Strauss et al., 2020), epidermal cell shapes (Sapala et al., 2018), leaf shape emergence (Runions et al., 2017), virtual crop generation (Marshall-Colon et al., 2017), auxin-driven patterning (Cieslak et al., 2015), control of bud activation (Prusinkiewicz et al., 2009), apical hook formation (Žádníková et al., 2016), fruit expansion (Cieslak et al., 2016), and inflorescence (Owens et al., 2016). L-systems can integrate external stimulus (i.e., temperature effect) and allow the prediction of plant phenotypes (Palubicki et al., 2009). Recent studies have also shown that L-systems can be combined with stochastic simulation algorithms to overcome typical limitations of purely deterministic model description (Cieslak and Prusinkiewicz, 2019). Furthermore, L-systems were combined with deep learning for the robust image processing of organ structures (Ubbens et al., 2018).

### The Cell Complex

One feature peculiar to plants is that cells cannot move with respect to one another and the only event affecting tissue topology is cell division. The “cell complex” is a recently developed system capable of capturing the topology of plant (Prusinkiewicz and Lane, 2013). The “cell complex” is defined by mathematical elements named “cells” (not to be confused with biological cells) of different topological dimensions (i.e., 0 for vertices, 1 for edges, 2 for faces, and 3 for volumes), all of them organized into coherent structures called “flip tables.” The flip tables are sufficient to represent the whole system, and they provide all the basic working operations such as iterating, merging, splitting, getting geometric information (orientation, boundaries), and so forth (Figure 3D). This representation is used to build the Cell Complex Framework, a C++ API which can be used for computational modeling in 2D and 3D. This API is part of the advanced 4D modeling framework MorphoDynamX.^1^ The cell complex is a recent innovation and therefore its applications are still scarce; nonetheless, models using cell complexes have been applied to heterocyst formation in *Anabaena* and leaf margin morphogenesis (Prusinkiewicz and Lane, 2013) as well as to cell division in the *Arabidopsis* embryo (Yoshida et al., 2014). At the same time, using the cell complex it was possible to recreate the complete 4D map of early *Arabidopsis* embryo development including all successive cell division events (Yoshida et al., 2014). Similarly, the cell complex was chosen as the base structure for the simulation of root emergence and development in a comprehensive model combining mechanical and biochemical signals (Marconi et al., 2021).

In summary, we reviewed some popular methods used to represent cells and tissues, focusing on modeling plant development. The next step is to extend such biological representations with the specific rules applied to organ growth and biomechanics.

## Cell Growth Modeling Approaches

After choosing the underlying digital representation of the plant organ one may apply a system of growth rules that allows the initial structure to evolve and generate the biological patterns found in nature. The majority of growth models focus on approximating the physical laws of movement. Organ growth can be coordinated either on global or local level; by defining general rules of organ development, or by specifying local rules for the individual elements composing the organ (i.e., individual cells). The accuracy of the representation largely depends both on the level of abstraction and the complexity of the physical rules. General biological models usually employ compact implementations that may simplify all the nuances of the physical laws of motion to provide qualitative and more universal solutions. In contrast, more specific or focused models dive into the mechanistic description of the underlying organ growth processes.

### Descriptive Growth Rules

**Figure 4:**
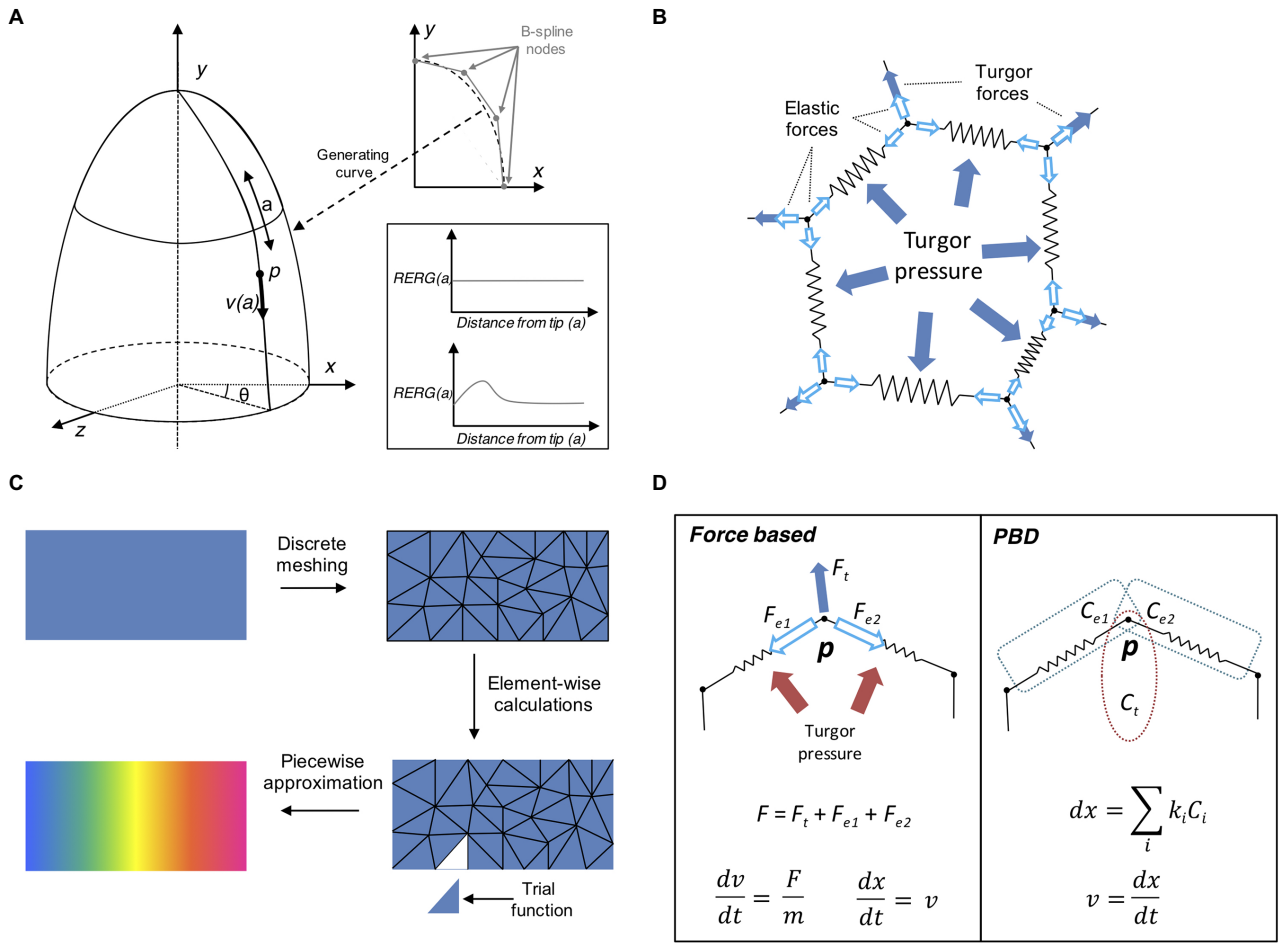
Computational approaches to tissue growth dynamics. **(A)** A descriptive model of growth the shoot apex (adapted from Smith et al., 2006b). The shoot surface layer can be generated by a B-spline rotating around the longitudinal axis of the shoot apex (upper right plot). A point *p* located on the surface of the apex is located at coordinates (*θ, a*) moves away from the apex tip with the velocity *v(a)*, where *a* is the distance from the apex tip, measured along the curve on the apex surface. The shape of the generating curve and the angle *θ* determines the growth in Cartesian coordinates. The two sample plots show the relative elemental rate of growth (RERG) as functions of distance from the apex tip, either at constant growth (lower right box, upper plot) or with a decreased growth rate at the apex tip and flank (lower right box, lower plot). **(B)** Schematic of a cell represented by the mass–spring system. The cell wall is a polygon whose vertices and edges are masses and springs, respectively. The effect of internal turgor pressure generates forces perpendicular to the wall edges, and distributed over the vertex masses. The vertices movement is further restricted by the elastic forces exerted by the springs. **(C)** Schematic representation of the finite element method (FEM). A continuous object for which a global function of growth needs to be approximated is subdivided into a conglomerate of smaller geometrically manageable finite elements (triangles in this case) in a process called “meshing.” Each element is represented by a set of piecewise linear equations (sometimes called “trial” functions) derived from the original problem. After the calculations are done for each finite element all sets of equations are systematically recombined into a global system that approximates the original one. Generally, a finer meshing results in a better approximation. **(D)** Comparison between the force- based approach and position based dynamics (PBD). In a force based approaches the total cumulative force is calculated for each physical entity. For instance, a mass–spring system includes the total force acting on the vertex *via* internal turgor pressure and the elastic forces of the springs. Acceleration and velocity are calculated for each time step (usually recurring to explicit or implicit integrating solvers), and the object position is finally updated. In the PBD approach, the object is subjected to different constraints instead (in this case, two distance constraints and an area constraint, see Müller et al., 2007; Marconi et al., 2021), and each constraint is sequentially projected on the object updating its position. The vertex velocity is recalculated in the final step.

In general, it is possible to achieve complex organ shapes by specifying global regulation for the entire organ or alternatively by determining local rules at cellular level. Local growth can be difficult to define as it requires controlling mechanical interactions between local entities to avoid conflicts and breaking the laws of physics (Rodriguez et al., 1994). A common approach at solving this problem is to use descriptive models of growth (Mosca et al., 2018). This type of models is sometimes called “organ-centric system,” as growth is accomplished by transforming plant tissues along a determined path in an Eulerian fashion. Organs that display a simple conserved geometrical shape can be represented with a descriptive growth model, as cell movement can be indirectly achieved by moving the cell along a path pre-defined by a mathematical function (Figure 4A). For example, the *Arabidopsis* root tip presents a radially symmetric geometrical shape resembling an elongated cylinder with a smoothed end (Dolan et al., 1993). Hejnowicz and Karczewski (1993) developed a descriptive model for root growth that is specified in linear parametric coordinates and later mapped onto the curvilinear Cartesian coordinates, this allows to easily reconstruct the root tip curvature. A similar idea has been applied to the plant shoot apex (Kucypera et al., 2017), by mapping polar coordinates to Cartesian coordinates. A major advantage of organ centric systems is that growth can be specified in only one dimension, thereby greatly reducing system complexity.

### Mass–Spring Systems

In a classical mass–spring system, the edges representing the cell membrane/wall are idealized mechanical springs each connected by two masses constituted by vertices (Figure 4B; Mosca et al., 2018). Internal forces caused by the cytoskeleton are sometimes ignored as they are much weaker compared to the forces exerted on the cell wall. A damping force is commonly applied to the masses to prevent numerical oscillations around the steady state. Springs usually have negligible mass, while the mass of vertices depends on the properties of the underlying tissue. Spring can be modeled as elastic, viscoelastic, or plastic elements, depending on the nature of the biological material. For instance, tissue expansion in vertex-based models relies on internal turgor pressure, which acts on the vertices masses of the cell membrane forcing the springs to elongate, while tissue growth can be easily achieved by updating the spring resting length. Mass–spring are arguably the simplest mechanical systems to implement and have been used to model the shoot apex (Hamant et al., 2008; Uyttewaal et al., 2012), petal shape (Rolland-Lagan et al., 2003), leaf venation networks (Corson et al., 2009a), shoot apical meristem (Corson et al., 2009b), tip-growing cells (Dumais et al., 2006), shoot apex cytoskeleton (Hamant et al., 2008), and cell–cell interaction (Dupuy et al., 2008), root development (Waidmann et al., 2019), and apical hook bending (Žádníková et al., 2016). A major limitation of the mass spring system is its dependency on mesh discretization, as alternative meshes may produce dramatically different results. Moreover, complex mechanical processes such as anisotropy are challenging to implement with these systems (Mosca et al., 2018).

### FEM Models

Finite element method (Becker et al., 1982) is a popular modeling technique borrowed from the engineering field and often used for modeling continuous mechanics of plant development. For many complex problems involving partial differential equations (PDE), the analytic solution is often not available, and it is necessary to recur to numerical methods to solve it. FEMs discretize a continuous mechanical problem to derive it efficiently over two or three space dimensions. Solving a FEM requires several steps that define how physical properties are applied onto a predefined geometrical structure (Figure 4C). First, we define the initial geometry of the system, created either from segmented sample images or manually drawn using dedicated computer tools (Bassel and Smith, 2016). The geometry is later subdivided into small discrete elements by constructing a polyhedral mesh of the object of interest. Each element is defined as interconnected nodes and the global behavior of the system is constrained by boundary conditions imposed on the structure. The modeler then applies external forces and defines the interactions between the elements of the system. The mechanical rules describe how the elements behave under forces including stresses and tension (Hamant et al., 2008).

Applying FEMs to model pavement cells revealed the local distribution of mechanical stresses (Sampathkumar et al., 2014). The cell wall is also another candidate system to be studied with FEMs, for example, by simulating the effect of turgor pressure over different wall shapes and thickness (Forouzesh et al., 2013). Tip growth of pollen tubes has been studied through FEM models as a hollow shell with uniform thickness (Fayant et al., 2010; Vogler et al., 2013), or to quantify data from micro-indentation (Bolduc et al., 2006). Similarly, FEMs were employed to simulate the branching morphogenesis of *Arabidopsis* trichomes (Yanagisawa et al., 2015), revealing a strong axial growth caused by the transversal alignment of microtubules. In the context of shape deformation, FEMs were applied to reconstruct the reversible shape changes of stomata guard cells of leaves pores (Bidhendi and Geitmann, 2018), to describe the mechanical feedback restricting sepal growth and shape (Hervieux et al., 2016), and the emergence of epidermal cell shapes (Sapala et al., 2018). Robinson and Kuhlemeier (2018) constructed a finite element model to understand the effect of external stresses on the hypocotyl, while a study of Bassel et al. (2014) recapitulated the mechanical outgrowth of an *Arabidopsis* root radicle. At a higher structural level, FEMs have been used to explain the modality of explosive seed dispersal (Hofhuis et al., 2016).

A powerful feature of FEM modeling is the integration of quantitative experimental data. Live cell imaging of the shoot apical meristem combined with finite-element modeling allowed to unravel the functional distinction between differential growing tissues beyond the sheer genetic specification (Kierzkowski et al., 2012). FEMs can also be combined with other modeling techniques; Fozard et al. (2013) (Jensen and Fozard, 2015) proposed a hybrid mass–spring/FEM “vertex-element” model by merging a vertex-based structure with a finite-element discretization of in-plane walls; while Boudon et al. (2015) succeed in applying FEMs to mechanical models of young meristems at “pseudo”-cellular resolution. Finally, understanding the relationship between gene activity and organ shaping led to the development of third-party computer applications such as GFtbox (Kennaway et al., 2011), which aims at integrating different components of plant morphogenesis into a coherent software interface based on FEM.

Overall, finite element modeling is a powerful tool that allows researchers to simulate the behavior of complex plant organ shapes and their interactions with genetic activity and environmental stresses; however, at the cost of high computational demand (as the organ grows) and (usually) lack of single-cell definition.

### Position Based Dynamics

Force-based systems (such as FEM and mass–spring models) represent the typical approach to deal with the mechanical properties of plant systems. Under this paradigm and Newton’s second law of motion, acceleration is computed from total internal and external forces. An explicit or implicit time integration method is then used to update the velocities and the positions of the mesh elements. Unfortunately, force-based systems are often unstable and require slow implicit integration to converge, which is a major limitation for modeling complex organ structures at cellular resolution.

Physically-based animation has always been a major topic in computer graphics (Bargteil et al., 2020). This field is concerned with finding new methods for the simulation of physical phenomena such as rigid body dynamics, objects deformation or fluid flow, with a strong focus on stability, speed, and robustness. Position based dynamics (PBD) is a recent physically-based animation technique that prevents the typical instability problems of force–based systems through the action of local constraints (Müller et al., 2007). With this approach it is possible to omit the velocity layer and immediately work on positions, making the system more stable and controllable (Figure 4D). PBD has been applied in a large number of applications, mainly outside the biological world (Bender et al., 2017). Our own study (Marconi et al., 2021) has shown its potential benefits in plant morphogenesis by implementing PBD inside the “cell complex” environment (Prusinkiewicz and Lane, 2013) and using it to model the growth or the *Arabidopsis* root (Marconi et al., 2021). Similar to other modeling techniques developed for physically-based animation, PBD provides higher performance and stability, thanks to the aforementioned replacement of force-based motion with *ad-hoc* designed constraint functions. However, as expected, such advantages come at the price; Newtonian forces are abstracted through constraints and therefore the dynamics of the system do not have a direct physical interpretation (Müller et al., 2007). Another limitation of PBD is its dependency on time step size and number of iterations (Bender et al., 2017). To address these problems, an alternative formulation of PBD has been derived by introducing a new constraint formulation that corresponds to a well-defined concept of elastic potential energy, which allows for solving constraints in a time step and iteration count independent manner (Macklin et al., 2016).

Taken together, modeling of organ growth is a complex endeavor as it requires avoiding conflicts between moving elements such as cells or tissues, while preserving the global structure of the organ. However, combining growth mechanics with biochemical reactions represents an increased level of complexity and is currently an active research subject.

## Survey of Mechanical and Biochemical Modeling Frameworks

The applications of computational techniques in biology have fueled the development of extendable frameworks to avoid the multiplication of models tailored for just a single problem, providing a fully integrated environment for computational modeling and hypothesis testing (Routier-Kierzkowska and Runions, 2018). In many instances, these frameworks can provide the user with a fully operational toolkit that abstracts the underlying implementation and allow the user to focus the effort on addressing the actual biological question. Several software packages have been developed to satisfy different needs for plant-related scientific problems, and most of them offer open-source licenses.

L-studio is a Microsoft Windows software that uses L-system (discussed above) to simulate models of plant morphogenesis; the corresponding version for Linux is known as Vlab (Karwowski and Prusinkiewicz, 2004). This modeling package represents one of the oldest software specifically designed for plant structure modeling. L-studio objects are C++ modules that can be loaded and executed into the main simulator. The simulator produces a visual representation of the model using the OpenGL graphics library.^2^ L-studio also provides a browser for organizing and accessing objects such as plant segments, cells, or tree branches on local and remote machines as well as a series of editors and other modeling tools for creating and modifying these objects.

OpenAlea is an user-friendly open-source platform that provides the researcher with a graphical user interface comprising a set of tools specifically dedicated to plant tissue modeling (Pradal et al., 2008). OpenAlea was designed to be an easy to use, reusable and extendable collaborative environment. The main advantage of this software is the definition of the model using a graphical language. Model components can be visually edited by the user and connected to other components. Each component contains rules and parameters that biologically define the execution of the model. Users can add new functionalities to the system as Python^3^ scripts through the package manager at run-time without modification of the framework. The current package includes a number of ready-to-use external modules like the VPlants package for plant architecture analysis and the PlantGL graphic library for plant geometric modeling. OpenAlea has been used in a number of models to describe processes ranging from auxin transport to root branching (Lucas et al., 2008; Perrine-Walker et al., 2010; Rutzinger et al., 2011).

VirtualLeaf is a cell-based computer modeling framework designed for plant tissue morphogenesis (Merks et al., 2011). This program allows to model cells, cell walls, chemicals diffusion, and reactions, and to define rules that regulate growth and development. VirtualLeaf inner mechanism is similar to the previously described CPM. One of the main motivations that justified the creation of this software library was the observation that plant cells are not allowed to slide along each other but are instead constrained by the presence of the cell wall. Common CPMs cannot prevent this issue, while VirtualLeaf offers an alternative off-lattice method that implements a working solution. VirtualLeaf has been successfully used to aid understanding plant morphogenesis in several different contexts (Wabnik et al., 2010; van Mourik et al., 2012; De Rybel et al., 2014; Qi et al., 2017).

Multicellular organisms are capable of producing extremely different shapes coordinated by interaction between individual cells. Cells communicate by different means through the exchange of mechanical and chemical signals. The desire to explicitly represent this network of interactions has driven the creation of CellModeller (Dupuy et al., 2008). CellModeller is a generic software tool designed for the analysis and modeling of plant morphogenesis at cellular resolution. This framework can execute systems with more than 1,000 cells and their interactions. Moreover, it is specifically designed for plant tissues, and it can be easily expanded with additional models using the XML file format (Dupuy et al., 2008).

The popular finite element method (described in the previous section) is the mathematical background for the GPT-framework (Kennaway et al., 2011). GFtbox, is a MATLAB application devised for flat organs commonly such as leaf and petal, but it is also compatible with other organ forms. Different patterns of growth can influence the deformation of continuous sheets of biological tissues creating vastly dissimilar shapes. The GFtbox can combine mechanical growth with gene activity, tissue identity, and biochemical properties at the organ level. For example, the GFtbox has been successfully implemented to describe growing polarized tissues and genetic control of morphogenetic processes (Kuchen et al., 2012; Sauret-Güeto et al., 2013).

MorphoGraphX is one of the most popular tools for visualization and analysis of 4D biological datasets (de Reuille et al., 2015). It allows the user to extract organ/cell shape through segmentation and to quantify cell growth and signal fluorescence. Moreover, this software allows the dynamic modeling of templates extracted from imaging data through simple scripting. MorphoGraphX can be easily expanded with external plugins. One of them is MorphoRobotX, an add-on used to analyze data from the cellular force microscope, a custom microindentation system that is specialized for plants (Routier-Kierzkowska et al., 2012). Recently, the new-generation version of MorphoGraphX called MorphoDynamX has been constructed based on the “cell complex” data structure described in the previous section (Prusinkiewicz and Lane, 2013). This addition greatly improves the opportunities of modeling subdividing geometry in 2 and 3 dimensions, a big step forward in comparison with simpler vertex-to-vertex technologies. MorphoDynamX and the cell complex are very recent developments but they have already shown their potential in the modeling of cell division in the *Arabidopsis* embryo (Yoshida et al., 2014) and the organogenesis of *Arabidopsis* root (Marconi et al., 2021). MorphoDynamX can also be expanded with external modules; for example, MorphoMechanX is an add-on for MorphoDynamX that enables the mechanical modeling of biological tissues using FEMs for solids elements (Long et al., 2020; Natonik-Białoń et al., 2020; Läubli et al., 2021).

Outside of plant world other frameworks could be adapted to model plant organs such as CompuCell3D (Cickovski et al., 2005) – a lattice-based multicellular modeling environment to simulate tissue formation by merging the CPM with chemical fields and diffusion equations. CompuCell3D functionalities have been demonstrated in the context of animal embryogenesis, cell population dynamics, tumor formation, and many more (Andasari et al., 2012; Swat et al., 2012; Fortuna et al., 2020; Liu et al., 2021). Another valuable tool to simulate animal embryogenesis is MecaGen, a C++ simulation platform of animal multicellular development relying on mechanistic agent-based models (Delile et al., 2017). This software is capable of combining cell mechanics with gene expression and intracellular signaling. For example, this tool has been applied to replicate zebrafish epiboly collective cell behavior (Delile et al., 2017), epithelialization of Drosophila wings (Neto-Silva et al., 2009), and cell fate specification in animal tissues (Chan et al., 2017).

To date, diverse computer modeling frameworks for plant organogenesis have been developed that typically integrate either biochemical or mechanical cues. These frameworks were critical in providing answers for many important scientific questions that otherwise could not be addressed with purely experimental approaches. However, there is still a shortage of user-friendly model frameworks that combine mechanics with gene regulation and intracellular signaling at single cell level, thus leaving a vast space for future improvements.

## Conclusion and Perspectives

Nowadays, computer models are a fundamental tool in virtually any field of scientific research. In plant science, the quantitative representation of organs and tissues still remains computationally challenging, in particular given the amount and quality of data needed to resolve growth at the cellular level. Moreover, constructing advanced models of morphogenesis requires the researchers to be competent not only in experimental techniques but also to possess a basic knowledge of programming, computer graphics, physics, mechanics, and biostatistics. Still, the potential benefits of including computer models into experimental research environments are vast; many are the cases where naive intuition about a specific natural process falls short, since deterministic consequences and causal connections that appear logically obvious can in fact be dangerously misleading. Computer simulations have demonstrated that complex forms can emerge from the dynamic interactions between minimal components of the system. Therefore, models allow researchers to separate the inquiry from his cognitive bias and to test hypotheses in an objective and reproducible way.

However, we must be extremely careful when interpreting and reporting the results obtained from computer simulations. Just like any other analytical approach, models are subject to the evergreen motto “garbage in, garbage out”; by feeding the model with erroneous, biased or imprecise data, we can at most expect the same quality as output. There are other common mistakes in computer modeling, related to the model definition: extremely simple/complex models that tend to underfit/overfit the data, low-definition graphical representations of the biological structure, unrealistic physical or chemical properties regulating system development, lack of practical use and self-fulfilling expectations fueled by bias reinforcement, among others. A strict collaboration between computer scientists and wet-lab researchers is of paramount importance to guarantee the biological relevance of computer simulations. Additionally, a continuous feedback between the model and experimental data ensures that the necessary assumptions and simplifications built into the model do not compromise the final inference.

In the area of development biology, these complications are further exacerbated by the intrinsic sequential nature of the subject under scrutiny: growth occurs over time and often specific developmental stages are hard to observe or completely unknown, this produces holes in the idealized mechanistic process that need to be carefully filled in accordance with biologically sound principles. Recognizing the role of space-time interactions and the importance of biomechanics in plant growth is fundamental to achieve a realistic simulation, as modeling can be expressed in 1D, 2D, and 3D (+ time). The modelers must also decide which level of abstraction should be considered; this means, for example, choosing whether to represent the tissue as a continuous material rather than subdividing it into single cells or other type of discrete representation. Depending on the process under investigation either approach can be more appropriate than the other.

The recent years have witnessed the development of a plethora of computational tools designed to unravel a disparate range of problems in plant morphogenesis. Nonetheless, there still remain many unanswered questions; what kind of feedback exists between cell division and mechanical/molecular processes? How do cells regulate their own anisotropic growth? What kinds of forces determine cell polarity (e.g., how are hormones transported and how are they distributed in a polar manner)? How does the cell wall expand under internal turgor pressure but withstand external forces? How do organs achieve a desired shape without the aid of global long-range signals? These are just a few open questions of which we possess only a partial knowledge, many others laid unanswered. We have the feeling that the future of computational modeling is bright and these models will yet play an essential important role in deciphering the mysteries of plant morphogenesis.

## Author Contributions

MM and KW wrote the entire manuscript with no additional contribution. All authors contributed to the article and approved the submitted version.

## Funding

This work was supported by the Programa de Atraccion de Talento 2017 (Comunidad de Madrid, 2017-T1/BIO-5654 to KW), Severo Ochoa (SO) Programme for Centres of Excellence in R&D from the Agencia Estatal de Investigacion of Spain [grant SEV-2016-0672 (2017–2021) to KW *via* the CBGP]. In the frame of SEV-2016-0672 funding MM is supported with a postdoctoral contract. KW was supported by Programa Estatal de Generacion del Conocimiento y Fortalecimiento Cientıfico y Tecnologico del Sistema de I+D+I 2019 (PGC2018-093387-A407 I00) from MICIU (to KW).

## Conflict of Interest

The authors declare that the research was conducted in the absence of any commercial or financial relationships that could be construed as a potential conflict of interest.

## Publisher’s Note

All claims expressed in this article are solely those of the authors and do not necessarily represent those of their affiliated organizations, or those of the publisher, the editors and the reviewers. Any product that may be evaluated in this article, or claim that may be made by its manufacturer, is not guaranteed or endorsed by the publisher.

## Figures and Tables

**Table 1:** 

Agent-based model	A computational model where the behavior of the system is determined by the actions and the interaction of the individual elements of the system.
Cellular automata	An abstract computational system used to general complex discrete patterns starting from simple units, called “cells” or “atoms.” The cells exist over a grid, representing the two-dimensional space over which the cells can move. Each cell possesses a state defining its current properties which can evolve according to some pre-defined rules.
Deep learning	A branch of machine learning concerned with the use of artificial neural network composed of several layers of complexity.
Eulerian	A coordinate system where particles are not observed independently but instead considered as a uniform fluid whose properties evolves as a function of time and space. This approach requires restricting the environment into a defined space by imposing boundary lines, and observing the motion of particles inside the space according to the laws of fluids motion.
Feedback-loop	A portion of a system where the output of an element of the system is used as input of the same element for future operations.
Formal grammar	An abstract structure that describes a formal language in a rigorous way, by setting the mathematical rules that defined the generation of complex symbols starting from a simple alphabet.
Lagrangian	A coordinates system where the motion of each particle is followed independently. The position and velocity of each particle and its reaction toward the environment is observed at each time point.
Lattice	A graph of interconnected vertices embedded into the Euclidean plane forming a regular tiling resembling a grid of points.
Mesh	In solid modeling, a mesh is a way to represent a polyhedral object using a set of vertices, edges, and faces.
Remeshing	The process of changing the structure of a mesh by completely redefining its topology or by modifying individual elements.
Steepest descent minimization	An iterative optimization algorithm used to find a local minimum of a differentiable function.
Topology	A branch of mathematics concerned with the properties of geometric objects and their deformations.
Viscoelastic	A material that can behave both elastically (returning to its original state after deformation) or plastically (partially conserving the state achieved after deformation).
Voronoi	A diagram obtained by subdividing a Euclidean plane into convex polygons using a set of generating points such that each polygon contains exactly one generating point and every point in a given polygon is closer to its generating point than to any other.
Yield	In mechanics, the yield point indicates the limit on a stress–strain curve above which we observe the end of elastic behavior and the onset of plastic behavior.

## Footnotes

^1^ https://www.mpipz.mpg.de/MorphoGraphX/MorphoDynamX

^2^ https://www.opengl.org

^3^ www.python.org

## References

[ref1] Abley K. Barbier de Reuille P. Strutt D. Bangham A. Prusinkiewicz P. Marée A. F. M.. (2013). An intracellular partitioning-based framework for tissue cell polarity in plants and animals. Development 140, 2061–2074. doi: 10.1242/dev.062984, PMID: 23633507

[ref2] Adler I. Barabe D. Jean R. V. (1997). A history of the study of phyllotaxis. Ann. Bot. 80, 231–244. doi: 10.1006/anbo.1997.0422

[ref3] Allen M. T. Prusinkiewicz P. DeJong T. M. (2005). Using L-systems for modeling source-sink interactions, architecture and physiology of growing trees: the L-PEACH model. New Phytol. 166, 869–880. doi: 10.1111/j.1469-8137.2005.01348.x, PMID: 15869648

[ref4] Alt S. Ganguly P. Salbreux G. (2017). Vertex models: from cell mechanics to tissue morphogenesis. Philos. Trans. R. Soc. Lond. Ser. B Biol. Sci. 372:20150520. doi: 10.1098/rstb.2015.0520, PMID: 28348254 PMC5379026

[ref5] Andasari V. Roper R. T. Swat M. H. Chaplain M. A. J. (2012). Integrating intracellular dynamics using CompuCell3D and bionetsolver: applications to multiscale modelling of cancer cell growth and invasion. PLoS One 7:e33726. doi: 10.1371/journal.pone.0033726, PMID: 22461894 PMC3312894

[ref6] Banwarth-Kuhn M. Nematbakhsh A. Rodriguez K. W. Snipes S. Rasmussen C. G. Reddy G. V.. (2019). Cell-based model of the generation and maintenance of the shape and structure of the multilayered shoot apical meristem of *Arabidopsis thaliana*. Bull. Math. Biol. 81, 3245–3281. doi: 10.1007/s11538-018-00547-z, PMID: 30552627

[ref7] Bargteil A. W. Shinar T. Kry P. G. (2020). An introduction to physics-based animation, In SIGGRAPH Asia 2020 Courses, SA 2020.

[ref8] Baskin T. I. (2005). Anisotropic expansion of the plant cell wall. Annu. Rev. Cell Dev. Biol. 21, 203–222. doi: 10.1146/annurev.cellbio.20.082503.103053, PMID: 16212493

[ref9] Bassel G. W. Smith R. S. (2016). Quantifying morphogenesis in plants in 4D. Curr. Opin. Plant Biol. 29, 87–94. doi: 10.1016/j.pbi.2015.11.005, PMID: 26748353

[ref10] Bassel G. W. Stamm P. Mosca G. De Reuille P. B. Gibbs D. J. Winter R.. (2014). Mechanical constraints imposed by 3D cellular geometry and arrangement modulate growth patterns in the *Arabidopsis* embryo. Proc. Natl. Acad. Sci. U. S. A. 111, 8685–8690. doi: 10.1073/pnas.1404616111, PMID: 24912195 PMC4060677

[ref11] Bayer E. M. Smith R. S. Mandel T. Nakayama N. Sauer M. Prusinkiewicz P.. (2009). Integration of transport-based models for phyllotaxis and midvein formation. Genes Dev. 23, 373–384. doi: 10.1101/gad.497009, PMID: 19204121 PMC2648550

[ref12] Becker E. B. Carey G. F. Oden J. T. Belytschko T. (1982). Finite elements, an introduction. J. Appl. Mech. 49:682. doi: 10.1115/1.3162562

[ref13] Bender J. Muller M. Macklin M. (2017). A survey on position based dynamics. Anal. Biochem. 6, 1–31. doi: 10.2312/egt.20171034

[ref14] Besson S. Dumais J. (2011). Universal rule for the symmetric division of plant cells. Proc. Natl. Acad. Sci. U. S. A. 108, 6294–6299. doi: 10.1073/pnas.1011866108, PMID: 21383128 PMC3076879

[ref15] Bidhendi A. J. Geitmann A. (2018). Finite element modeling of shape changes in plant cells. Plant Physiol. 176, 41–56. doi: 10.1104/pp.17.01684, PMID: 29229695 PMC5761827

[ref16] Bolduc J. F. Lewis L. J. Aubin C. É. Geitmann A. (2006). Finite-element analysis of geometrical factors in micro-indentation of pollen tubes. Biomech. Model. Mechanobiol. 5, 227–236. doi: 10.1007/s10237-005-0010-1, PMID: 16514520

[ref17] Boudon F. Chopard J. Ali O. Gilles B. Hamant O. Boudaoud A.. (2015). A computational framework for 3D mechanical modeling of plant morphogenesis with cellular resolution. PLoS Comput. Biol. 11:e1003950. doi: 10.1371/journal.pcbi.1003950, PMID: 25569615 PMC4288716

[ref18] Chakrabortty B. Blilou I. Scheres B. Mulder B. M. (2018). A computational framework for cortical microtubule dynamics in realistically shaped plant cells. PLoS Comput. Biol. 14:e1005959. doi: 10.1371/journal.pcbi.1005959, PMID: 29394250 PMC5812663

[ref19] Chan C. J. Heisenberg C. P. Hiiragi T. (2017). Coordination of morphogenesis and cell-fate specification in development. Curr. Biol. 27, R1024–R1035. doi: 10.1016/j.cub.2017.07.010, PMID: 28950087

[ref20] Cickovski T. M. Huang C. Chaturvedi R. Glimm T. Hentschel H. G. E. Alber M. S.. (2005). A framework for three-dimensional simulation of morphogenesis. IEEE/ACM Trans. Comput. Biol. Bioinform. 2, 273–288. doi: 10.1109/TCBB.2005.46, PMID: 17044166

[ref21] Cieslak M. Cheddadi I. Boudon F. Baldazzi V. Génard M. Godin C.. (2016). Integrating physiology and architecture in models of fruit expansion. Front. Plant Sci. 7:1739. doi: 10.3389/fpls.2016.01739, PMID: 27917187 PMC5116533

[ref22] Cieslak M. Prusinkiewicz P. (2019). Gillespie-lindenmayer systems for stochastic simulation of morphogenesis. In Silico Plants 1. doi: 10.1093/insilicoplants/diz009

[ref23] Cieslak M. Runions A. Prusinkiewicz P. (2015). Auxin-driven patterning with unidirectional fluxes. J. Exp. Bot. 66, 5083–5102. doi: 10.1093/jxb/erv262, PMID: 26116915 PMC4513925

[ref24] Cohen D. (1967). Computer simulation of biological pattern generation processes. Nature 216, 246–248. doi: 10.1038/216246a0, PMID: 6053826

[ref25] Corson F. Adda-Bedia M. Boudaoud A. (2009a). In silico leaf venation networks: growth and reorganization driven by mechanical forces. J. Theor. Biol. 259, 440–448. doi: 10.1016/j.jtbi.2009.05.002, PMID: 19446571

[ref26] Corson F. Hamant O. Bohn S. Traas J. Boudaoud A. Couder Y. (2009b). Turning a plant tissue into a living cell froth through isotropic growth. Proc. Natl. Acad. Sci. U. S. A. 106, 8453–8458. doi: 10.1073/pnas.0812493106, PMID: 19423667 PMC2688973

[ref27] Cosgrove D. (1986). Biophysical control of plant cell growth. Annu. Rev. Plant Physiol. 37, 377–405. doi: 10.1146/annurev.pp.37.060186.002113, PMID: 11539701

[ref28] Cosgrove D. J. (1993). Water uptake by growing cells: an assessment of the controlling roles of wall relaxation, solute uptake, and hydraulic conductance. Int. J. Plant Sci. 154, 10–21. doi: 10.1086/297087, PMID: 11537965

[ref29] Cosgrove D. J. (2005). Growth of the plant cell wall. Nat. Rev. Mol. Cell Biol. 6, 850–861. doi: 10.1038/nrm1746, PMID: 16261190

[ref30] Cosgrove D. J. (2016). Plant cell wall extensibility: connecting plant cell growth with cell wall structure, mechanics, and the action of wall-modifying enzymes. J. Exp. Bot. 67, 463–476. doi: 10.1093/jxb/erv511, PMID: 26608646

[ref31] Davidson L. A. Joshi S. D. Kim H. Y. von Dassow M. Zhang L. Zhou J. (2010). Emergent morphogenesis: elastic mechanics of a self-deforming tissue. J. Biomech. 43, 63–70. doi: 10.1016/j.jbiomech.2009.09.010, PMID: 19815213 PMC2813421

[ref32] de Boer M. J. M. Fracchia F. D. Prusinkiewicz P. (1992). “A model for cellular development in morphogenetic fields,” in Lindenmayer Systems: Impacts on Theoretical Computer Science, Computer Graphics, and Developmental Biology (Berlin, Heidelberg: Springer-Verlag), 351–370.

[ref33] de Keijzer J. Mulder B. M. Janson M. E. (2014). Microtubule networks for plant cell division. Syst. Synth. Biol. 8, 187–194. doi: 10.1007/s11693-014-9142-x, PMID: 25136380 PMC4127175

[ref34] de Reuille P. B. Routier-Kierzkowska A. L. Kierzkowski D. Bassel G. W. Schüpbach T. Tauriello G.. (2015). MorphoGraphX: a platform for quantifying morphogenesis in 4D. elife 4, 1–20. doi: 10.7554/eLife.05864, PMID: 25946108 PMC4421794

[ref35] De Rybel B. Adibi M. Breda A. S. Wendrich J. R. Smit M. E. Novák O.. (2014). Integration of growth and patterning during vascular tissue formation in *Arabidopsis*. Science 345:1255215. doi: 10.1126/science.1255215, PMID: 25104393

[ref36] Deinum E. E. Tindemans S. H. Lindeboom J. J. Mulder B. M. (2017). How selective severing by katanin promotes order in the plant cortical microtubule array. Proc. Natl. Acad. Sci. U. S. A. 114, 6942–6947. doi: 10.1073/pnas.1702650114, PMID: 28630321 PMC5502621

[ref37] Delile J. Herrmann M. Peyriéras N. Doursat R. (2017). A cell-based computational model of early embryogenesis coupling mechanical behaviour and gene regulation. Nat. Commun. 8:13929. doi: 10.1038/ncomms13929, PMID: 28112150 PMC5264012

[ref38] Dinneny J. R. (2020). Mechanobiology: plant cells face pressure from neighbors. Curr. Biol. 30, R344–R346. doi: 10.1016/j.cub.2020.02.025, PMID: 32315631

[ref39] Dolan L. Janmaat K. Willemsen V. Linstead P. Poethig S. Roberts K.. (1993). Cellular organisation of the *Arabidopsis thaliana* root. Development 119, 71–84. doi: 10.1242/dev.119.1.71, PMID: 8275865

[ref40] Dong J. MacAlister C. A. Bergmann D. C. (2009). BASL controls asymmetric cell division in *Arabidopsis*. Cell 137, 1320–1330. doi: 10.1016/j.cell.2009.04.018, PMID: 19523675 PMC4105981

[ref41] Dumais J. Shaw S. L. Steele C. R. Long S. R. Ray P. M. (2006). An anisotropic-viscoplastic model of plant cell morphogenesis by tip growth. Int. J. Dev. Biol. 50, 209–222. doi: 10.1387/ijdb.052066jd, PMID: 16479489

[ref42] Dupuy L. Mackenzie J. Haseloff J. (2010). Coordination of plant cell division and expansion in a simple morphogenetic system. Proc. Natl. Acad. Sci. U. S. A. 107, 2711–2716. doi: 10.1073/pnas.0906322107, PMID: 20133808 PMC2823878

[ref43] Dupuy L. MacKenzie J. Rudge T. Haseloff J. (2008). A system for modelling cell-cell interactions during plant morphogenesis. Ann. Bot. 101, 1255–1265. doi: 10.1093/aob/mcm235, PMID: 17921524 PMC2710276

[ref01] Eden M. (1961). “A two-dimensional growth process.” in Proceedings of the fourth Berkeley symposium on mathematical statistics and probability (Berkeley: University of California Press), 223–239.

[ref44] Errera L. (1888). Uber zellformen und seifenblasen. Bot. Zentralbl. 34, 395–398.

[ref45] Fayant P. Girlanda O. Chebli Y. Aubin C. É. Villemure I. Geitmann A. (2010). Finite element model of polar growth in pollen tubes. Plant Cell 22, 2579–2593. doi: 10.1105/tpc.110.075754, PMID: 20699395 PMC2947179

[ref46] Fletcher A. G. Osterfield M. Baker R. E. Shvartsman S. Y. (2014). Vertex models of epithelial morphogenesis. Biophys. J. 106, 2291–2304. doi: 10.1016/j.bpj.2013.11.4498, PMID: 24896108 PMC4052277

[ref47] Forouzesh E. Goel A. MacKenzie S. A. Turner J. A. (2013). In vivo extraction of *Arabidopsis* cell turgor pressure using nanoindentation in conjunction with finite element modeling. Plant J. 73, 509–520. doi: 10.1111/tpj.12042, PMID: 23036157

[ref48] Fortuna I. Perrone G. C. Krug M. S. Susin E. Belmonte J. M. Thomas G. L.. (2020). CompuCell3D simulations reproduce mesenchymal cell migration on flat substrates. Biophys. J. 118, 2801–2815. doi: 10.1016/j.bpj.2020.04.024, PMID: 32407685 PMC7264849

[ref49] Fozard J. A. Lucas M. King J. R. Jensen O. E. (2013). Vertex-element models for anisotropic growth of elongated plant organs. Front. Plant Sci. 4:233. doi: 10.3389/fpls.2013.00233, PMID: 23847638 PMC3706750

[ref50] Garnett P. Steinacher A. Stepney S. Clayton R. Leyser O. (2010). Computer simulation: the imaginary friend of auxin transport biology. Bioessays 32, 828–835. doi: 10.1002/bies.200900185, PMID: 20652891

[ref51] Geitmann A. Ortega J. K. E. (2009). Mechanics and modeling of plant cell growth. Trends Plant Sci. 14, 467–478. doi: 10.1016/j.tplants.2009.07.006, PMID: 19717328

[ref52] Glazier J. A. Graner F. (1993). Simulation of the differential adhesion driven rearrangement of biological cells. Phys. Rev. E Stat. Phys. Plasmas Fluids Relat. Interdiscip. Topics 47, 2128–2154. doi: 10.1103/PhysRevE.47.2128, PMID: 9960234

[ref53] Grieneisen V. A. Xu J. Marée A. F. M. Hogeweg P. Scheres B. (2007). Auxin transport is sufficient to generate a maximum and gradient guiding root growth. Nature 449, 1008–1013. doi: 10.1038/nature06215, PMID: 17960234

[ref54] Hamant O. Heisler M. G. Jönsson H. Krupinski P. Uyttewaal M. Bokov P.. (2008). Developmental patterning by mechanical signals in *Arabidopsis*. Science 322, 1650–1655. doi: 10.1126/science.1165594, PMID: 19074340

[ref55] Hamant O. Inoue D. Bouchez D. Dumais J. Mjolsness E. (2019). Are microtubules tension sensors? Nat. Commun. 10:2360. doi: 10.1038/s41467-019-10207-y, PMID: 31142740 PMC6541610

[ref56] Hartmann A. Fischer D. Kinzel L. Chowdhury S. P. Hofmann A. Baldani J. I.. (2019). Assessment of the structural and functional diversities of plant microbiota: achievements and challenges—a review. J. Adv. Res. 19, 3–13. doi: 10.1016/j.jare.2019.04.007, PMID: 31341665 PMC6629839

[ref57] Hejnowicz Z. Karczewski J. (1993). Modeling of meristematic growth of root apices in a natural coordinate system. Am. J. Bot. 80, 309–315. doi: 10.1002/j.1537-2197.1993.tb13804.x

[ref58] Hejnowicz Z. Romberger J. A. (1984). Growth tensor of plant organs. J. Theor. Biol. 110, 93–114. doi: 10.1016/S0022-5193(84)80017-X

[ref59] Hejnowicz Z. Rusin A. Rusin T. (2000). Tensile tissue stress affects the orientation of cortical microtubules in the epidermis of sunflower hypocotyl. J. Plant Growth Regul. 19, 31–44. doi: 10.1007/s003440000005, PMID: 11010990

[ref60] Hervieux N. Dumond M. Sapala A. Routier-Kierzkowska A. L. Kierzkowski D. Roeder A. H. K.. (2016). A mechanical feedback restricts sepal growth and shape in *Arabidopsis*. Curr. Biol. 26, 1019–1028. doi: 10.1016/j.cub.2016.03.004, PMID: 27151660

[ref61] Hofhuis H. Moulton D. Lessinnes T. Routier-Kierzkowska A. L. Bomphrey R. J. J. Mosca G.. (2016). Morphomechanical innovation drives explosive seed dispersal. Cell 166, 222–233. doi: 10.1016/j.cell.2016.05.002, PMID: 27264605 PMC4930488

[ref62] Honda H. (1971). Description of the form of trees by the parameters of the tree-like body: effects of the branching angle and the branch length on the shape of the tree-like body. J. Theor. Biol. 31, 331–338. doi: 10.1016/0022-5193(71)90191-3, PMID: 5557081

[ref63] Honda H. (1983). Geometrical models for cells in tissues. Int. Rev. Cytol. 81, 191–248. doi: 10.1016/S0074-7696(08)62339-6, PMID: 6347934

[ref64] Hong L. Dumond M. Zhu M. Tsugawa S. Li C. B. Boudaoud A.. (2018). Heterogeneity and robustness in plant morphogenesis: from cells to organs. Annu. Rev. Plant Biol. 69, 469–495. doi: 10.1146/annurev-arplant-042817-040517, PMID: 29505739

[ref65] Hwang M. Garbey M. Berceli S. A. Tran-Son-Tay R. (2009). Rule-based simulation of multi-cellular biological systems-a review of modeling techniques. Cell. Mol. Bioeng. 2, 285–294. doi: 10.1007/s12195-009-0078-2, PMID: 21369345 PMC3045734

[ref66] Jensen O. E. Fozard J. A. (2015). Multiscale models in the biomechanics of plant growth. Physiology 30, 159–166. doi: 10.1152/physiol.00030.2014, PMID: 25729061 PMC4346705

[ref67] Jönsson H. Heisler M. G. Shapiro B. E. Meyerowitz E. M. Mjolsness E. (2006). An auxin-driven polarized transport model for phyllotaxis. Proc. Natl. Acad. Sci. U. S. A. 103, 1633–1638. doi: 10.1073/pnas.0509839103, PMID: 16415160 PMC1326488

[ref68] Jürgens G. (2001). Apical-basal pattern formation in *Arabidopsis* embryogenesis. EMBO J. 20, 3609–3616. doi: 10.1093/emboj/20.14.3609, PMID: 11447101 PMC125542

[ref69] Karwowski R. Prusinkiewicz P. (2004). “The L-system-based plant-modeling environment L-studio 4.0,” in *Proceedings of the 4th international workshop on functional-structural plant models;* June 7–11, 2004; Montpellier, France.

[ref70] Kennaway R. Coen E. Green A. Bangham A. (2011). Generation of diverse biological forms through combinatorial interactions between tissue polarity and growth. PLoS Comput. Biol. 7:e1002071. doi: 10.1371/journal.pcbi.1002071, PMID: 21698124 PMC3116900

[ref71] Kepinski S. Leyser O. (2005). Plant development: auxin in loops. Curr. Biol. 15, R208–R210. doi: 10.1016/j.cub.2005.03.012, PMID: 15797014

[ref72] Kierzkowski D. Nakayama N. Routier-Kierzkowska A. L. Weber A. Bayer E. Schorderet M.. (2012). Elastic domains regulate growth and organogenesis in the plant shoot apical meristem. Science 335, 1096–1099. doi: 10.1126/science.1213100, PMID: 22383847

[ref73] Kierzkowski D. Routier-Kierzkowska A. L. (2019). Cellular basis of growth in plants: geometry matters. Curr. Opin. Plant Biol. 47, 56–63. doi: 10.1016/j.pbi.2018.09.008, PMID: 30308452

[ref74] Kierzkowski D. Runions A. Vuolo F. Strauss S. Lymbouridou R. Routier-Kierzkowska A. L.. (2019). A growth-based framework for leaf shape development and diversity. Cell 177, 1405.e17–1418.e17. doi: 10.1016/j.cell.2019.05.011, PMID: 31130379 PMC6548024

[ref75] Korn R. W. (1969). A stochastic approach to the development of coleocheate. J. Theor. Biol. 24, 147–158. doi: 10.1016/S0022-5193(69)80042-1, PMID: 5822650

[ref76] Krul T. Kaandorp J. A. Blom J. G. (2003). “Modelling developmental regulatory networks,” in Lecture Notes in Computer Science. ICCS 2003. Vol. 2660. eds. P. M. A. Sloot, D. Abramson, A. V. Bogdanov, Y. E. Gorbachev, J. J. Dongarra and A. Y. Zomaya (Berlin, Heidelberg: Springer), 688–697.

[ref77] Kuchen E. E. Fox S. De Reuille P. B. Kennaway R. Bensmihen S. Avondo J.. (2012). Generation of leaf shape through early patterns of growth and tissue polarity. Science 335, 1092–1096. doi: 10.1126/science.1214678, PMID: 22383846

[ref78] Kucypera K. Lipowczan M. Piekarska-Stachowiak A. Nakielski J. (2017). A method to generate the surface cell layer of the 3D virtual shoot apex from apical initials. Plant Methods 13:110. doi: 10.1186/s13007-017-0262-7, PMID: 29238397 PMC5725887

[ref79] Laskowski M. Grieneisen V. A. Hofhuis H. Ten Hove C. A. Hogeweg P. Marée A. F. M.. (2008). Root system architecture from coupling cell shape to auxin transport. PLoS Biol. 6:e307. doi: 10.1371/journal.pbio.0060307, PMID: 19090618 PMC2602721

[ref80] Läubli N. F. Burri J. T. Marquard J. Vogler H. Mosca G. Vertti-Quintero N.. (2021). 3D mechanical characterization of single cells and small organisms using acoustic manipulation and force microscopy. Nat. Commun. 12:2583. doi: 10.1038/s41467-021-22718-8, PMID: 33972516 PMC8110787

[ref81] Leyser O. (2018). Auxin signaling. Plant Physiol. 176, 465–479. doi: 10.1104/pp.17.00765, PMID: 28818861 PMC5761761

[ref82] Li S. Bashline L. Lei L. Gu Y. (2014). Cellulose synthesis and its regulation. Arabidopsis Book 12:e0169. doi: 10.1199/tab.0169, PMID: 24465174 PMC3894906

[ref83] Liepman A. H. Wightman R. Geshi N. Turner S. R. Scheller H. V. (2010). *Arabidopsis*—a powerful model system for plant cell wall research. Plant J. 61, 1107–1121. doi: 10.1111/j.1365-313X.2010.04161.x, PMID: 20409281

[ref84] Lindenmayer A. (1968). Mathematical models for cellular interactions in development I. filaments with one-sided inputs. J. Theor. Biol. 18, 280–299. doi: 10.1016/0022-5193(68)90079-9, PMID: 5659071

[ref85] Lindenmayer A. (1975). Developmental algorithms for multicellular organisms: a survey of L-systems. J. Theor. Biol. 54, 3–22. doi: 10.1016/S0022-5193(75)80051-8, PMID: 1202291

[ref86] Lindenmayer A. (1977). Paracladial relationships in leaves. Ber. Dtsch. Bot. Ges. 90, 287–301. doi: 10.1111/j.1438-8677.1977.tb02822.x

[ref87] Liu R. Higley K. A. Swat M. H. Chaplain M. A. J. Powathil G. G. Glazier J. A.. (2021). Development of a coupled simulation toolkit for computational radiation biology based on Geant4 and CompuCell3D. Phys. Med. Biol. 66:045026. doi: 10.1088/1361-6560/ac1f37, PMID: 33339019

[ref88] Lockhart J. A. (1965). An analysis of irreversible plant cell elongation. J. Theor. Biol. 8, 264–275. doi: 10.1016/0022-5193(65)90077-9, PMID: 5876240

[ref89] Long Y. Cheddadi I. Mosca G. Mirabet V. Dumond M. Kiss A.. (2020). Cellular heterogeneity in pressure and growth emerges from tissue topology and geometry. Curr. Biol. 30, 1504.e8–1516.e8. doi: 10.1016/j.cub.2020.02.027, PMID: 32169211

[ref90] Louveaux M. Julien J. D. Mirabet V. Boudaoud A. Hamant O. (2016). Cell division plane orientation based on tensile stress in *Arabidopsis thaliana*. Proc. Natl. Acad. Sci. U. S. A. 113, E4294–E4303. doi: 10.1073/pnas.1600677113, PMID: 27436908 PMC4968720

[ref91] Lucas M. Guédon Y. Jay-Allemand C. Godin C. Laplaze L. (2008). An auxin transport-based model of root branching in *Arabidopsis thaliana*. PLoS One 3:e3673. doi: 10.1371/journal.pone.0003673, PMID: 18989371 PMC2577305

[ref92] MacAl C. M. North M. J. (2010). Tutorial on agent-based modelling and simulation. J. Simul. 4, 151–162. doi: 10.1057/jos.2010.3

[ref93] Macklin M. Müller M. Chentanez N. (2016). “XPBD: Position-based simulation of compliant constrained dynamics,” in *Proceedings—Motion in Games 2016: 9th International Conference on Motion in Games*, MIG 2016, 49–54.

[ref94] Mähönen A. P. Tusscher K. Ten Siligato R. Smetana O. Díaz-Triviño S. Salojärvi J.. (2014). PLETHORA gradient formation mechanism separates auxin responses. Nature 515, 125–129. doi: 10.1038/nature13663, PMID: 25156253 PMC4326657

[ref95] Mansfield C. Newman J. L. Olsson T. S. G. Hartley M. Chan J. Coen E. (2018). Ectopic BASL reveals tissue cell polarity throughout leaf development in *Arabidopsis thaliana*. Curr. Biol. 28, 2638.e4–2646.e4. doi: 10.1016/j.cub.2018.06.019, PMID: 30100337 PMC6109230

[ref96] Marconi M. Gallemi M. Benková E. Wabnik K. (2021). A coupled mechano-biochemical framework for root meristem morphogenesis. bioRxiv [Preprint]. doi: 10.1101/2021.01.27.428294 PMC8716106 34723798

[ref97] Marshall-Colon A. Long S. P. Allen D. K. Allen G. Beard D. A. Benes B.. (2017). Crops in silico: generating virtual crops using an integrative and multi-scale modeling platform. Front. Plant Sci. 8:786. doi: 10.3389/fpls.2017.00786, PMID: 28555150 PMC5430029

[ref98] Masson P. H. Tasaka M. Morita M. T. Guan C. Chen R. Boonsirichai K. (2002). *Arabidopsis thaliana*: a model for the study of root and shoot gravitropism. Arabidopsis Book 1:e0043. doi: 10.1199/tab.0043, PMID: 22303208 PMC3243349

[ref99] Mele B. H. Giannino F. Vincenot C. E. Mazzoleni S. Cartení F. (2015). Cell-based models in plant developmental biology: insights into hybrid approaches. Front. Environ. Sci. 3:73. doi: 10.3389/fenvs.2015.00073

[ref100] Merks R. M. H. Guravage M. Inzé D. Beemster G. T. S. (2011). Virtualleaf: an open-source framework for cell-based modeling of plant tissue growth and development. Plant Physiol. 155, 656–666. doi: 10.1104/pp.110.167619, PMID: 21148415 PMC3032457

[ref101] Mirabet V. Krupinski P. Hamant O. Meyerowitz E. M. Jönsson H. Boudaoud A. (2018). The self-organization of plant microtubules inside the cell volume yields their cortical localization, stable alignment, and sensitivity to external cues. PLoS Comput. Biol. 14:e1006011. doi: 10.1371/journal.pcbi.1006011, PMID: 29462151 PMC5834207

[ref102] Mironova V. V. Omelyanchuk N. A. Yosiphon G. Fadeev S. I. Kolchanov N. A. Mjolsness E.. (2010). A plausible mechanism for auxin patterning along the developing root. BMC Syst. Biol. 4:98. doi: 10.1186/1752-0509-4-98, PMID: 20663170 PMC2921385

[ref103] Mitchison G. J. (1977). Phyllotaxis and the fibonacci series. Science 196, 270–275. doi: 10.1126/science.196.4287.270, PMID: 17756084

[ref104] Mosca G. Adibi M. Strauss S. Runions A. Sapala A. Smith R. S. (2018). “Modeling plant tissue growth and cell division,” in Mathematical Modelling in Plant Biology. ed. Morris R. (Cham: Springer), 107–138.

[ref105] Moukhtar J. Trubuil A. Belcram K. Legland D. Khadir Z. Urbain A.. (2019). Cell geometry determines symmetric and asymmetric division plane selection in *Arabidopsis* early embryos. PLoS Comput. Biol. 15:e1006771. doi: 10.1371/journal.pcbi.1006771, PMID: 30742612 PMC6386405

[ref106] Müller M. Heidelberger B. Hennix M. Ratcliff J. (2007). Position based dynamics. J. Vis. Commun. Image Represent. 18, 109–118. doi: 10.1016/j.jvcir.2007.01.005

[ref107] Natonik-Białoń S. Borowska-Wykręt D. Mosca G. Grelowski M. Wrzalik R. Smith R. S.. (2020). Deformation of a cell monolayer due to osmotic treatment: a case study of onion scale epidermis. Botany 98, 21–36. doi: 10.1139/cjb-2019-0027

[ref108] Neto-Silva R. M. Wells B. S. Johnston L. A. (2009). Mechanisms of growth and homeostasis in the Drosophila wing. Annu. Rev. Cell Dev. Biol. 25, 197–220. doi: 10.1146/annurev.cellbio.24.110707.175242, PMID: 19575645 PMC2760035

[ref109] Niklas K. J. (1977). Applications of finite element analyses to problems in plant morphology. Ann. Bot. 41, 133–153. doi: 10.1093/oxfordjournals.aob.a085261

[ref110] Ortega J. K. E. (1985). Augmented growth equation for cell wall expansion. Plant Physiol. 79, 318–320. doi: 10.1104/pp.79.1.318, PMID: 16664396 PMC1074876

[ref111] Ötvös K. Marconi M. Vega A. Brien J. O. Johnson A. Abualia R.. (2021). Modulation of root growth by nutrient-defined regulation of polar auxin transport. EMBO J. 40. doi: 10.15252/embj.2020106862, PMID: 33399250 PMC7849315

[ref112] Owens A. Cieslak M. Hart J. Classen-Bockhoff R. Prusinkiewicz P. (2016). Modeling dense inflorescences. ACM Trans. Graph. 35, 1–14. doi: 10.1145/2897824.2925982

[ref113] Palubicki W. Horel K. Longay S. Runions A. Lane B. Měch R.. (2009). Self-organizing tree models for image synthesis. ACM Trans. Graph. 28, 1–10. doi: 10.1145/1531326.1531364

[ref114] Paradez A. Wright A. Ehrhardt D. W. (2006). Microtubule cortical array organization and plant cell morphogenesis. Curr. Opin. Plant Biol. 9, 571–578. doi: 10.1016/j.pbi.2006.09.005, PMID: 17010658

[ref115] Perianez-Rodriguez J. Rodriguez M. Marconi M. Bustillo-Avendaño E. Wachsman G. Sanchez-Corrionero A.. (2021). An auxin-regulable oscillatory circuit drives the root clock in *Arabidopsis*. Sci. Adv. 7:eabd4722. doi: 10.1126/sciadv.abd4722, PMID: 33523850 PMC7775764

[ref116] Perrine-Walker F. Doumas P. Lucas M. Vaissayre V. Beauchemin N. J. Band L. R.. (2010). Auxin carriers localization drives auxin accumulation in plant cells infected by frankia in casuarina glauca actinorhizal nodules. Plant Physiol. 154, 1372–1380. doi: 10.1104/pp.110.163394, PMID: 20826704 PMC2971613

[ref117] Peters W. S. Tomos A. D. (1996). The history of tissue tension. Ann. Bot. 77, 657–665. doi: 10.1093/aob/77.6.657, PMID: 11541099

[ref118] Petricka J. J. Winter C. M. Benfey P. N. (2012). Control of *Arabidopsis* root development. Annu. Rev. Plant Biol. 63, 563–590. doi: 10.1146/annurev-arplant-042811-105501, PMID: 22404466 PMC3646660

[ref119] Pradal C. Dufour-Kowalski S. Boudon F. Fournier C. Godin C. (2008). OpenAlea: a visual programming and component-based software platform for plant modelling. Funct. Plant Biol. 35, 751–760. doi: 10.1071/FP08084, PMID: 32688829

[ref120] Proseus T. E. Ortega J. K. E. Boyer J. S. (1999). Separating growth from elastic deformation during cell enlargement. Plant Physiol. 119, 775–784. doi: 10.1104/pp.119.2.775, PMID: 9952474 PMC32155

[ref121] Prusinkiewicz P. Cieslak M. Ferraro P. Hanan J. (2018). “Modeling plant development with L-systems,” in Mathematical Modelling in Plant Biology. ed. Morris R. (Cham: Springer), 139–169.

[ref122] Prusinkiewicz P. Crawford S. Smith R. S. Ljung K. Bennett T. Ongaro V.. (2009). Control of bud activation by an auxin transport switch. Proc. Natl. Acad. Sci. U. S. A. 106, 17431–17436. doi: 10.1073/pnas.0906696106, PMID: 19805140 PMC2751654

[ref123] Prusinkiewicz P. Karwowski R. Mĕch R. Hanan J. (2000). “L-studio/cpfg: a software system for modeling plants,” in Applications of Graph Transformations with Industrial Relevance. AGTIVE 1999. Lecture Notes in Computer Science. Vol. 1779. eds. Nagl M. Schürr A. Münch M. (Berlin, Heidelberg: Springer), 457–464.

[ref124] Prusinkiewicz P. Lane B. (2013). “Modeling morphogenesis in multicellular structures with cell complexes and L-systems,” in Pattern Formation in Morphogenesis: Springer Proceedings in Mathematics. *Vol* 15. eds. Capasso V. Gromov M. Harel-Bellan A. Morozova N. Pritchard L. (Berlin, Heidelberg: Springer), 137–151.

[ref125] Prusinkiewicz P. Runions A. (2012). Computational models of plant development and form. New Phytol. 193, 549–569. doi: 10.1111/j.1469-8137.2011.04009.x, PMID: 22235985

[ref126] Qi J. Wu B. Feng S. Lü S. Guan C. Zhang X.. (2017). Mechanical regulation of organ asymmetry in leaves. Nat. Plants 3, 724–733. doi: 10.1038/s41477-017-0008-6, PMID: 29150691

[ref127] Rayle D. L. Cleland R. E. (1992). The acid growth theory of auxin-induced cell elongation is alive and well. Plant Physiol. 99, 1271–1274. doi: 10.1104/pp.99.4.1271, PMID: 11537886 PMC1080619

[ref128] Robbins N. E. Dinneny J. R. (2018). Growth is required for perception of water availability to pattern root branches in plants. Proc. Natl. Acad. Sci. U. S. A. 115, E822–E831. doi: 10.1073/pnas.1710709115, PMID: 29317538 PMC5789911

[ref129] Robinson S. Kuhlemeier C. (2018). Global compression reorients cortical microtubules in *Arabidopsis* hypocotyl epidermis and promotes growth. Curr. Biol. 28, 1794.e2–1802.e2. doi: 10.1016/j.cub.2018.04.028, PMID: 29804811

[ref130] Rodriguez E. K. Hoger A. McCulloch A. D. (1994). Stress-dependent finite growth in soft elastic tissues. J. Biomech. 27, 455–467. doi: 10.1016/0021-9290(94)90021-3, PMID: 8188726

[ref131] Rojas E. R. Huang K. C. (2018). Regulation of microbial growth by turgor pressure. Curr. Opin. Microbiol. 42, 62–70. doi: 10.1016/j.mib.2017.10.015, PMID: 29125939

[ref132] Rolland-Lagan A. G. Bangham J. A. Coen E. (2003). Growth dynamics underlying petal shape and asymmetry. Nature 422, 161–163. doi: 10.1038/nature01443, PMID: 12634785

[ref133] Rolland-Lagan A. G. Coen E. Impey S. J. Bangham J. A. (2005). A computational method for inferring growth parameters and shape changes during development based on clonal analysis. J. Theor. Biol. 232, 157–177. doi: 10.1016/j.jtbi.2004.04.045, PMID: 15530487

[ref134] Routier-Kierzkowska A. L. K. Runions A. (2018). “Modeling plant morphogenesis: An introduction,” in Plant Biomechanics: From Structure to Function at Multiple Scales. eds. Geitmann A. Gril J. (Cham: Springer), 165–192.

[ref135] Routier-Kierzkowska A. L. Weber A. Kochova P. Felekis D. Nelson B. J. Kuhlemeier C.. (2012). Cellular force microscopy for in vivo measurements of plant tissue mechanics. Plant Physiol. 158, 1514–1522. doi: 10.1104/pp.111.191460, PMID: 22353572 PMC3343728

[ref136] Runions A. Fuhrer M. Lane B. Federl P. Rolland-Lagan A. G. Prusinkiewicz P. (2005). Modeling and visualization of leaf venation patterns. ACM Trans. Graph. 24, 702–711. doi: 10.1145/1073204.1073251

[ref137] Runions A. Tsiantis M. (2017). The shape of things to come: from typology to predictive models for leaf diversity. Am. J. Bot. 104, 1437–1441. doi: 10.3732/ajb.1700251, PMID: 29885230

[ref138] Runions A. Tsiantis M. Prusinkiewicz P. (2017). A common developmental program can produce diverse leaf shapes. New Phytol. 216, 401–418. doi: 10.1111/nph.14449, PMID: 28248421 PMC5638099

[ref139] Rutzinger M. Pratihast A. K. Elberink S. J. O. Vosselman G. (2011). Tree modelling from mobile laser scanning data-sets. Photogramm. Rec. 26, 361–372. doi: 10.1111/j.1477-9730.2011.00635.x

[ref140] Sahlin P. Jönsson H. (2010). A modeling study on how cell division affects properties of epithelial tissues under isotropic growth. PLoS One 5:e11750. doi: 10.1371/journal.pone.0011750, PMID: 20689588 PMC2912771

[ref141] Sampathkumar A. Krupinski P. Wightman R. Milani P. Berquand A. Boudaoud A.. (2014). Subcellular and supracellular mechanical stress prescribes cytoskeleton behavior in *Arabidopsis* cotyledon pavement cells. Elife 3:e01967. doi: 10.7554/elife.01967, PMID: 24740969 PMC3985187

[ref142] Sapala A. Runions A. Routier-Kierzkowska A. L. Gupta M. D. Hong L. Hofhuis H.. (2018). Why plants make puzzle cells, and how their shape emerges. Elife 7:e32794. doi: 10.7554/eLife.32794, PMID: 29482719 PMC5841943

[ref143] Sauret-Güeto S. Schiessl K. Bangham A. Sablowski R. Coen E. (2013). JAGGED controls *Arabidopsis* petal growth and shape by interacting with a divergent polarity field. PLoS Biol. 11:e1001550. doi: 10.1371/journal.pbio.1001550, PMID: 23653565 PMC3641185

[ref144] Schopfer P. (2006). Biomechanics of plant growth. Am. J. Bot. 93, 1415–1425. doi: 10.3732/ajb.93.10.1415, PMID: 21642088

[ref145] Shabala S. N. Lew R. R. (2002). Turgor regulation in osmotically stressed *Arabidopsis* epidermal root cells. Direct support for the role of inorganic ion uptake as revealed by concurrent flux and cell turgor measurements. Plant Physiol. 129, 290–299. doi: 10.1104/pp.020005, PMID: 12011359 PMC155892

[ref146] Silk W. K. Erickson R. O. (1979). Kinematics of plant growth. J. Theor. Biol. 76, 481–501. doi: 10.1016/0022-5193(79)90014-6, PMID: 439916

[ref147] Silk W. K. Wagner K. K. (1980). Growth-sustaining water potential distributions in the primary corn root. Plant Physiol. 66, 859–863. doi: 10.1104/pp.66.5.859, PMID: 16661542 PMC440742

[ref148] Smith C. (2006). On vertex-vertex systems and their use in geometric and biological modelling. Comput. Sci. doi: 10.11575/PRISM/439

[ref149] Smith R. S. Bayer E. M. (2009). Auxin transport-feedback models of patterning in plants. Plant Cell Environ. 32, 1258–1271. doi: 10.1111/j.1365-3040.2009.01997.x, PMID: 19453483

[ref150] Smith R. S. Guyomarc’h S. Mandel T. Reinhardt D. Kuhlemeier C. Prusinkiewicz P. (2006a). A plausible model of phyllotaxis. Proc. Natl. Acad. Sci. U. S. A. 103, 1301–1306. doi: 10.1073/pnas.0510457103, PMID: 16432192 PMC1345713

[ref151] Smith R. S. Kuhlemeier C. Prusinkiewicz P. (2006b). Inhibition fields for phyllotactic pattern formation: a simulation study. Can. J. Bot. 84, 1635–1649. doi: 10.1139/B06-133

[ref152] Smithers E. T. Luo J. Dyson R. J. (2019). Mathematical principles and models of plant growth mechanics: from cell wall dynamics to tissue morphogenesis. J. Exp. Bot. 70, 3587–3600. doi: 10.1093/jxb/erz253, PMID: 31128070

[ref153] Strauss S. Lempe J. Prusinkiewicz P. Tsiantis M. Smith R. S. (2020). Phyllotaxis: is the golden angle optimal for light capture? New Phytol. 225, 499–510. doi: 10.1111/nph.16040, PMID: 31254398

[ref154] Swat M. H. Thomas G. L. Belmonte J. M. Shirinifard A. Hmeljak D. Glazier J. A. (2012). Multi-scale modeling of tissues using CompuCell3D. Methods Cell Biol. 110, 325–366. doi: 10.1016/B978-0-12-388403-9.00013-8, PMID: 22482955 PMC3612985

[ref155] Ten Hove C. A. Lu K. J. Weijers D. (2015). Building a plant: cell fate specification in the early *Arabidopsis* embryo. Development 142, 420–430. doi: 10.1242/dev.111500, PMID: 25605778

[ref156] Thompson D. (1917). On Growth and Form. 1st *Edn*.

[ref157] Turing A. M. (1952). The chemical basis of morphogenesis. Philos. Trans. R. Soc. Lond. B. Biol. Sci. 237, 37–72. doi: 10.1098/rstb.1952.0012 PMC4360114 25750229

[ref158] Ubbens J. Cieslak M. Prusinkiewicz P. Stavness I. (2018). The use of plant models in deep learning: an application to leaf counting in rosette plants. Plant Methods 14:6. doi: 10.1186/s13007-018-0273-z, PMID: 29375647 PMC5773030

[ref159] Ulam S. (1962). On some mathematical problems connected with patterns of growth in figures. Math. Probl. Biol. Sci., 14, 215–224. doi: 10.1090/psapm/014/9947

[ref160] Uyttewaal M. Burian A. Alim K. Landrein B. Borowska-Wykrt D. Dedieu A.. (2012). Mechanical stress acts via Katanin to amplify differences in growth rate between adjacent cells in *Arabidopsis*. Cell 149, 439–451. doi: 10.1016/j.cell.2012.02.048, PMID: 22500806

[ref161] van Mourik S. Kaufmann K. van Dijk A. D. J. Angenent G. C. Merks R. M. H. Molenaar J. (2012). Simulation of organ patterning on the floral meristem using a polar auxin transport model. PLoS One 7:e28762. doi: 10.1371/journal.pone.0028762, PMID: 22291882 PMC3264561

[ref162] Van Norman J. M. (2016). Asymmetry and cell polarity in root development. Dev. Biol. 419, 165–174. doi: 10.1016/j.ydbio.2016.07.009, PMID: 27426272

[ref163] Van Opheusden J. H. J. Molenaar J. (2018). Algorithm for a particle-based growth model for plant tissues. R. Soc. Open Sci. 5:181127. doi: 10.1098/rsos.181127, PMID: 30564405 PMC6281936

[ref164] Veytsman B. A. Cosgrove D. J. (1998). A model of cell wall expansion based on thermodynamics of polymer networks. Biophys. J. 75, 2240–2250. doi: 10.1016/S0006-3495(98)77668-4, PMID: 9788919 PMC1299898

[ref165] Vlad D. Kierzkowski D. Rast M. I. Vuolo F. Dello Ioio R. Galinha C.. (2014). Leaf shape evolution through duplication, regulatory diversification and loss of a homeobox gene. Science 343, 780–783. doi: 10.1126/science.1248384, PMID: 24531971

[ref166] Vogler H. Draeger C. Weber A. Felekis D. Eichenberger C. Routier-Kierzkowska A. L.. (2013). The pollen tube: a soft shell with a hard core. Plant J. 73, 617–627. doi: 10.1111/tpj.12061, PMID: 23106269

[ref167] Voss-Böhme A. (2012). Multi-scale modeling in morphogenesis: a critical analysis of the cellular potts model. PLoS One 7:e42852. doi: 10.1371/journal.pone.0042852, PMID: 22984409 PMC3439478

[ref168] Wabnik K. Kleine-Vehn J. Balla J. Sauer M. Naramoto S. Reinöhl V.. (2010). Emergence of tissue polarization from synergy of intracellular and extracellular auxin signaling. Mol. Syst. Biol. 6:447. doi: 10.1038/msb.2010.103, PMID: 21179019 PMC3018162

[ref169] Wabnik K. Robert H. S. Smith R. S. Friml J. (2013). Modeling framework for the establishment of the apical-basal embryonic axis in plants. Curr. Biol. 23, 2513–2518. doi: 10.1016/j.cub.2013.10.038, PMID: 24291090

[ref170] Waidmann S. Rosquete M. R. Schöller M. Sarkel E. Lindner H. LaRue T.. (2019). Cytokinin functions as an asymmetric and anti-gravitropic signal in lateral roots. Nat. Commun. 10:3540. doi: 10.1038/s41467-019-11483-4, PMID: 31387989 PMC6684572

[ref171] Weliky M. Minsuk S. Keller R. Oster G. (1991). Notochord morphogenesis in Xenopus laevis: simulation of cell behavior underlying tissue convergence and extension. Development 113, 1231–1244. doi: 10.1242/dev.113.4.1231, PMID: 1811939

[ref172] Wolpert L. (1969). Positional information and the spatial pattern of cellular differentiation. J. Theor. Biol. 25, 1–47. doi: 10.1016/S0022-5193(69)80016-0, PMID: 4390734

[ref173] Yanagisawa M. Desyatova A. S. Belteton S. A. Mallery E. L. Turner J. A. Szymanski D. B. (2015). Patterning mechanisms of cytoskeletal and cell wall systems during leaf trichome morphogenesis. Nat. Plants 1:15014. doi: 10.1038/nplants.2015.14, PMID: 27246881

[ref174] Yoshida S. BarbierdeReuille P. Lane B. Bassel G. W. Prusinkiewicz P. Smith R. S.. (2014). Genetic control of plant development by overriding a geometric division rule. Dev. Cell 29, 75–87. doi: 10.1016/j.devcel.2014.02.002, PMID: 24684831

[ref175] Žádníková P. Wabnik K. Abuzeineh A. Gallemi M. Van Der Straeten D. Smith R. S.. (2016). A model of differential growth-guided apical hook formation in plants. Plant Cell 28, 2464–2477. doi: 10.1105/tpc.15.00569, PMID: 27754878 PMC5134968

[ref176] Zhang T. Cieslak M. Owens A. Wang F. Broholm S. K. Teeri T. H.. (2021). Phyllotactic patterning of gerbera flower heads. Proc. Natl. Acad. Sci. U. S. A. 118:e2016304118. doi: 10.1073/pnas.2016304118, PMID: 33771923 PMC8020676

